# Enhanced Detection of Organochlorine Pesticide Residues in Sesame Seeds (*Sesamum indicum L*.) Using Advanced GC‐MS/MS Techniques

**DOI:** 10.1155/jamc/8312847

**Published:** 2026-01-20

**Authors:** Kero Assefa Ago, Molla Tefera

**Affiliations:** ^1^ Department of Chemistry, Jinka University, P.O. Box 165, Jinka, South Ethiopia Region, Ethiopia; ^2^ Department of Chemistry, College of Natural Sciences, University of Gondar, P.O. Box 196, Gondar, Amhara, Ethiopia, uog.edu.et

**Keywords:** GC-MS/MS, organochlorine pesticides, QuEChERS, sesame seed

## Abstract

Considering the economic and edible values of sesame seeds, it is important to monitor sesame seed for the safety of consumers and for international trade as it helps the country government and suppliers as products to gain market acceptance more effectively. The objective of this study was to determine selected organochlorine pesticide (OCP) residues in sesame seeds. Quick, easy, cheap, efficient, rugged, and safe method followed by gas chromatography coupled with tandem mass spectrometry (GC‐MS/MS) was used for the rapid separation and determination of 20 OCPs in sesame seeds. Acetonitrile in combination with 1% glacial acetic acid was used as an extraction solvent. Primary secondary amine, graphitized carbon black, and octadecylsilane in QuEChERS kit were used for d‐SPE clean up before GC‐MS/MS analysis. The GC‐MS/MS analysis was evaluated in terms of linearity, recovery, and precision. The calibration curves were obtained for all analytes and displayed good linearity over the selected concentration range with regression coefficients (*r* ^2^) ≥ 0.999. The recoveries for spiked analytes in sesame seed samples were ranged from 93.58 to 115.81 with RSDs lower than 1%. The LOD and LOQ for all investigated pesticides were in the range of 0.05–0.88 μg/kg and from 0.16 to 2.93 μg/kg, respectively.

## 1. Introduction

Sesame seeds (*Sesamum indicum L*.) are a significant oilseed crop, valued for their nutritional properties, including high levels of healthy fats, protein, vitamins, minerals, and dietary fibers [1]. They are consumed directly as seeds or processed into oil, making them a staple in many diets worldwide. In terms of global total sesame production, Ethiopia ranked ninth in 2019 with an annual production of 2,62,654 tons, after Sudan, Myanmar, India, Tanzania, Nigeria, China, and China Mainland [2]. This encourages trend in production and export intensely from time to time.

However, the indiscriminate and repeated use of synthetic pesticides accumulates the toxic residues on agriculture that produce and pose a series threat to the health of consumers and international market [3]. Organochlorine pesticides (OCPs) are a class of synthetic chemicals widely used for agricultural pest control. This is due to their effectiveness and inexpensive in most of the developing environment [4].

OCPs include dichlorodiphenyltrichloroethane (DDT) and its metabolites, aldrin, and chlordane [5]. While effective in controlling pests, they are known for their potential to bioaccumulate in the food chain and pose significant health risks to humans and wildlife, including endocrine disruption and carcinogenic effects [6].

Due to rigorous measures adopted for international trade, the incidents of rejection of export assignments of food commodities due to pesticide residues have increasingly reported [7]. Like other agricultural products, sesame seeds can be contaminated with pesticides, which raise concerns about food safety and public health [8].

Considering the economic and edible values of sesame seeds, it is important to monitor sesame seed for safety of consumers and international trade. Regulatory bodies such as the Food and Agriculture Organization (FAO) and the World Health Organization (WHO) have established maximum residue limits (MRLs) for OCPs in food products [9]. Monitoring and ensuring that these limits are not exceeded is crucial for consumer safety.

However, the analysis of OCPs in oily seed matrices is challenging due to the large number of matrices in the compounds to be detected, the widespread physicochemical properties of the compounds (solubility, volatility, and polarity), the environmental conditions, the existence of pesticides at the trace level, lack of single standard technique for analysis and/or detection, and the foods to be tested are typically complex in nature [10].

Therefore, the detection and quantification of residues in complex matrices need sensitive and reliable analytical approaches. Gas chromatography coupled with tandem mass spectrometry (GC‐MS/MS) is one of the most advanced techniques available for this purpose. This technique separates compounds based on their volatility and interaction with the stationary phase in the column. OCPs, being volatile, are well suited for GC analysis. MS provides qualitative and quantitative data by measuring the mass‐to‐charge ratio of ions [11]. The tandem MS (MS/MS) capability enhances sensitivity and specificity by allowing for multiple stages of mass analysis, which is particularly useful for complex matrices like sesame seeds.

In chemical analysis, sample preparation steps involving extraction/isolation and/or preconcentration of analyte from a complex matrix is critical prior to their qualitative and/or quantitative determination [12]. Some of the most commonly employed methods are liquid–liquid extraction (LLE) [13] and solid‐phase extraction (SPE) [14]. Beside their advantages, the use of large volume of toxic organic solvents, generation of large volume of wastes, poor enrichment of analytes, and emulsion formation are considerable limitations [15].

On the other hand, quick, easy, cheap, effective, rugged, and safe (QuEChERS) is one of the pretreatment techniques that has gained incredible popularity among scholars during pesticide analysis in agricultural matrices [16]. Since its beginning, this method is considered particularly suitable and effective in the extraction of a wide range of compounds [17]. Consequently, most of the methods, developed using QuEChERS, involve extracting pesticides and contaminants from various food samples, particularly fruits, vegetables, and other food products [18]. The QuEChERS technique involves two steps: LLE and dispersive solid‐phase extraction (d‐SPE) cleanup [19]. The pretreated samples using QuEChERS are clean enough to be analyzed using gas chromatography.

The QuEChERS method in conjunction with GC/ECD [20], GC/NPD [21], or GC‐MS/MS [22] has been applied for the analysis of pesticide residues in different food matrices. About 16 OCPs in sesame seeds were determined by microwave‐assisted extraction and analyzed by GC/MS [23].

However, all the methods have their own shortcomings, including consumption of large amount of organic solvents, time, energy, and selectivity toward elemental composition. Moreover, most of analytical methods used for the determination of OCP residues are time consuming, expensive, requires confirmation, wasteful of organic solvents, and uses chlorinated solvents. To minimize these shortcomings, this study focuses on the use of nonchlorinated organic solvents, less time consuming, effective cheap, and easiest sample preparation technique with nonselective analytical methods. While numerous studies have examined OCP residues in various crops globally, this is one of the first comprehensive investigations specifically targeting sesame seeds from Metema, Ethiopia. This regional focus addresses a critical gap in the literature regarding food safety in Ethiopian agriculture. Therefore, the objective of this study was to investigate the presence of OCP residues in sesame seeds (*Sesamum indicum L*.) employing the AOAC QuEChERS method for sample preparation, providing an efficient cleanup process prior to GC‐MS/MS analysis.

## 2. Methodology

### 2.1. Description of Studying Area

**Figure 1:**
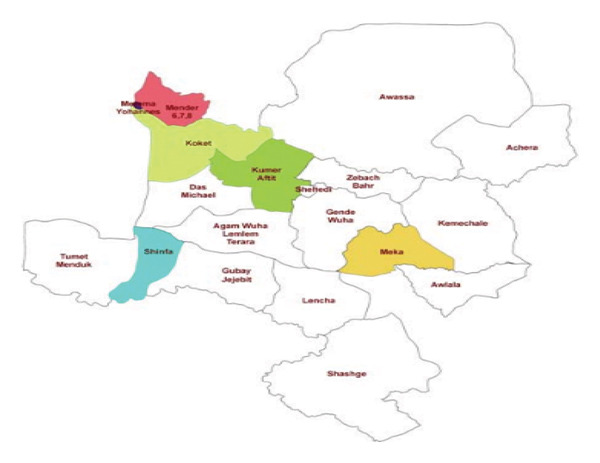
Sampling sites in Metema woreda.

The study was conducted at University of Gondar by collecting samples from Metema woreda, North Gondar, Ethiopia (Figure 1). The sampling site is located between 12°40′00″N and 36°8′00″E. It is 925 km North West of Addis Ababa and 187 km west of Gondar. The district has an international boundary of more than 60 km long distance between Ethiopia and Sudan. It contains 18 rural and 2 urban Kebels (ARDO, 2005). Its mean annual temperature ranges from 22°C to 28°C and daily temperature reaches as high as 43°C during the months of March–May. Mean annual rain fall ranges from 850 to 1100 mm and has unimodal pattern. The rainy months extend from June to the beginning of October. The most commonly grown crops in Metema woreda include sesame, cotton, and sorghum. Sesame took the largest share of the area. The total average cultivated land covered by sesame, cotton, and sorghum were 47%, 23%, and 26%, respectively [24].

### 2.2. Sample Collection

White sesame seed samples were collected from five different sites, namely, Kokit, Metema Yohan’s, Aftit, Das, and Mender 6.7.8 Kebeles (Figure 1). The collected samples were coded as BSC2683 and with Lot No. 2017‐MET‐05. The seed samples were cleaned and stored under a refrigerated at 4°C for one week before testing. To prevent contamination or degradation, seed samples were packed with airtight containers. Afterwards, the samples were homogenized and a portion of it was prepared for grinding into fine powders by grinder.

### 2.3. Instrument and Equipment

Electronic (analytical) balance (pyrx, England), grinder (type 20, Kika‐Wereke, German), centrifuge tubes, micropipettes (Dragon laboratory, China), vortex (Multi shaker) (Azer Scientific), and Agilent GC‐MS/MS (Aglient‐7000 GC‐TQ‐MS/MS, USA) equipped with a triple quadrupole inert mass selective detector (GC–2010 QP Mass Spectrometer) with an electron ionization chamber (70 eV) were used. A D‐B‐5MS1701 GC‐capillary columns (i.e., 30 m, 0.25 mm, and 0.25 μm) (Agilent technology, USA) were used. The injection was performed with an electronically controlled split/splitless injection port mode at 250 C and interfaced to the triple quadrupole inert mass selective detector. The injector temperature was set at 225°C through injection volume of 1.0 μL, and sample injection was performed directly in the splitless mode (1 min). The oven temperature was programmed as follows: initial temperature was set at 59°C held for 0.5 min ramped by 20°C/min at 150°C (0 min), raised to 170°C (20°C/min) for 1 min, raised to 224°C (54°C/min) for 1 min, elevated to 250°C (26°C/min) for 1 min, and raised to 270°C (20°/C min) for 1 min to separate OCPs with total run time of 30.5 min. High‐purity helium, over 99.999%, was used as carrier gas. The mass conditions were also set as follows: ionization mode with EI of ionization energy 70 eV, ion source temperature at 230°C, transfer line temperature at 280°C, and acquisition mode of multiple reaction monitoring (MRM) with positive polarity. The mass acquisition started at 4 min. For data acquisition and processing, Agilent enhanced mass transfer GC/MS data Acquisition work station software was used.

### 2.4. Chemicals and Reagents

Standard mixtures of 20 OCPs including Aldrin, α‐BHC, β‐BHC, γ‐BHC, δ‐BHC, lindane, α‐chlordane, γ‐chlordane, α‐endosulfan, β‐endosulfan, endosulfan sulfate, endrin, heptachlor, 4,4′‐DDD, 4,4′‐DDE, 4,4′‐DDT, dieldrin, endrin ketone, heptachlor exo‐epoxide, and methoxychlor with high purity ≥ 99% were purchased from Sigma‐Aldrich, USA, and decachlorobiphenyl (DCBP) (purity, 98.5% used as IS; Augsburg, Germany) was obtained from Bless Agri Food Laboratory Services PLC, Ethiopia. Acetonitrile purity of 99.9% analytical grade (Sigma‐Aldrich, USA), acetone 99% HPLC grade (Sigma‐Aldrich, USA), and glacial acetic acid purity of 99.83% (Morristown, NJ, USA) were used. Anhydrous magnesium sulfate (MgSO_4_) (anhydrous reagent plus ≥ 99.5%, Sigma Aldrich) and anhydrous sodium acetate with purity of 99% (Central drug House (P) Ltd, New Delhi, India) were applied. Mixtures of primary secondary amine (PSA), GCB, and C18 within a single QuEChERS kit (Sigma‐Aldrich, USA) were used for the cleanup process.

### 2.5. Stock and Standard Preparation

A 5.0‐mg solid mixture of pesticide standards was weighed for the preparation of stock standard solution and then transferred into 100‐mL volumetric flasks. Then, it was dissolved in acetonitrile for the preparation of 50.0 μg/mL solution. Intermediate and working standard solution were prepared with serious dilution of stock standard solution in acetonitrile from lower to higher concentration to minimize contamination. From stock solution, 1 μg/mL was taken for preparation of intermediate solutions. A 0.5 μg/mL solution was prepared by taking 50 mL of 1 mg/L intermediate solution and finally, 0.002, 0.005, 0.01, 0.02, 0.04, 0.15, and 0.25 μg/mL were prepared by diluting with acetonitrile for constructing calibration curves and preparation of the fortified sesame seed samples. Working standard solutions were stored at −20°C. Sesame seed samples were collected from supermarkets and sesame producers in Metema.

### 2.6. Sample Preparation

Sesame seed samples were bought from selected Kebeles of Metema and tested for the target list of analytes using GC‐MS/MS. To determine and validate the method further, the sample matrix was tested before spiking the known concentration of analytes to the sample solutions.

The blank matrix of dry sesame seed sample (0.5 kg) was homogeneously spiked with 10 mL of a mix‐standard solution (10 μg/mL). This mixture was kept standing for 45 min and then subjected to a thorough grinding using electronic grinder. Sesame seed was first homogenized, crushed, and mixed with water in a ratio of 1:2 (0.5 kg sample + 1 L water) with the guidance of literature [25].

### 2.7. QuEChERS Cleanup

This study deals with acetate buffered QuEChERS procedure with some modifications in which 1% acetic acid and sodium acetate in place of sodium chloride were used [26]. The use of 1% glacial acetic acid in combination with acetonitrile enhances the efficiency and effectiveness of the extraction process for pesticide residues in complex sesame seed samples. A 15.0 g of ground seed sample was transferred in to a 50‐mL Teflon centrifuge tube. Then, 15.0 mL of acetonitrile containing 1% acetic acid was added into centrifuge tube containing the ground sample. A 100.0‐μL DCBP was used as an internal standard to minimize matrix effect and added into the solution to ensure the accuracy of reported concentrations in GC‐MS/MS analysis. DCBP was selected as a surrogate internal standard due to its structural similarity to the target OCPs, ensuring comparable extraction efficiency and evaporation behavior. Its high chlorination provides a late retention time, preventing coelution with any target analytes or matrix components. Furthermore, DCBP is absent from analyzed samples and provides distinct, robust mass spectrometric signals in the MRM mode, enabling it to effectively correct for procedural losses and matrix‐induced variability throughout the analysis. The variations in the exact volume of the sample and the mixture were potted immediately and shaken by vortex for a minute. Afterwards, the mixed solution was followed by the addition of QuEChERS extraction salts (6.0:1.5 g) of MgSO_4_ and NaOAC for phase separation, respectively. Then, the whole mixture was vortexed for 1 min and the solution was transferred into 15‐mL Teflon centrifuge tube, which is better time usage than other reported method (3 min) [27]. It was then extracted by centrifugation at 4000 rpm for 5 min. Subsequently, 6.0 μL of clear solution was transferred into another new centrifuge tube through micropipette ranging 1–100 μL. A d‐SPE cleanup procedure with prepacked salt mixtures in the QuEChERS Kit (PSA, GCB, and C18) was used to remove color and lipid like interferences, then vortexed to avoid the salt crystalline agglomerates and to discard the color, and centrifuged at 4000 rpm for 5 min, and the upper clear solution was transferred to a new cleaned centrifuge tube by using micropipette. Then, after the cleared solution was filtered by using 0.45‐μm PTFE filter for GC‐MS/MS analysis without the need of evaporation; finally, 1.0 μL of filtered solution was automatically injected for GC‐MS/MS analysis.

### 2.8. QuEChERS‐Based Extraction

The QuEChERS approach has been shown to be effective in minimizing coextraction of lipids from fatty foods due to low solubility of the lipids in acetonitrile. Acetonitrile is commonly used as an extraction solvent in analytical chemistry, particularly for extracting organic compounds from food matrices, due to its excellent solvating properties and ability to dissolve a wide range of polar and nonpolar compounds. The addition of 1% glacial acetic acid serves several important purposes including pH adjustment, protonation of analytes, improved extraction efficiency, minimizing matrix effects, and compatibility with cleanup procedures and GC‐MS/MS techniques. Lipophilic analytes are partially recovered depending on the partitioning ratios and volumes of the acetonitrile. An acetonitrile solvent with 1% acetic acid was used for effective extractions of pesticides by minimizing potential interferences from samples. Through the results of the extracts of sesame seeds with acetonitrile, the extraction efficiencies were stable and indicated the preferable sample preparation technique.

## 3. Results and Discussion

### 3.1. Validation of the Method

The extraction procedure was validated in terms of linearity, recovery, limit of detection (LOD) and quantification (LOQ).

### 3.2. Linearity Study

To create calibration solutions, a blank matrix without any detectable residues of the analytes was utilized. An aliquot of this blank extract was then spiked with a specified quantity of pesticide mixture. We assessed linearity by developing calibration curve points with standard solutions in acetonitrile, ranging from 2.0 to 250.0 μg/mL. The calibration curves, which were derived from measurements of the chromatogram peak areas for each analyte, demonstrated excellent linearity across the chosen concentration range, exhibiting linear regression correlation coefficients (*r* ^2^) ≥ 0.999 as shown in Table 1 and Supporting Information (SI1a‐u).

**Table 1:** Analytical parameters of the QuEChERS–GC‐MS/MS method.

Pesticide	LR (μg·L^−1^)	*R* ^2^	LOD (μg/kg)	LOQ (μg/kg)	Intra‐day precision	Inter‐day precision
					10 (μg/kg)	10 (μg/kg)
Aldrin	1.80–250	0.9999	0.54	1.80	6.7	8.3
α‐BHC	2.06–250	0.9999	0.60	2.06	2.1	3.4
β‐BHC	2.46–250	0.9995	0.71	2.46	2.5	3.8
δ‐BHC	1.63–250	0.9997	0.49	1.63	2.8	5.1
Lindane	2.93–250	0.9998	0.88	2.93	4.9	7.7
α‐Chlordane	2.80–250	0.9998	0.84	2.80	2.2	5.6
γ‐Chlordane	1.06–250	0.9998	0.32	1.06	3.1	5.3
4,4′‐DDD	1.43–250	0.9996	0.49	1.43	5.9	8.2
4,4′‐DDT	2.30–250	0.9994	0.69	2.30	4.8	6.5
4,4′‐DDE	1.76–250	0.9999	0.53	1.76	8.3	9.1
Dieldrin	0.26–250	0.9999	0.08	0.26	2.6	3.1
Endosulfan sulfate (α‐ES)	2.00–250	0.9999	0.60	2.00	2.6	4.2
Endosulfan sulfate (β‐ES)	1.30–250	0.9997	0.39	1.30	2.9	3.1
Endosulfan sulfate (ESS)	2.03–250	0.9991	0.61	2.03	3.2	5.5
Endrin	1.70–250	0.9996	0.51	1.70	2.4	3.3
Endrin aldehyde (EA)	1.46–250	0.9994	0.44	1.46	2.1	2.4
Endrin ketone (EK)	0.26–250	0.9996	0.07	0.26	2.5	3.7
Heptachlor (HC)	0.16–250	0.9996	0.05	0.16	4.1	5.4
Heptachlor epoxide (HCE)	0.26–250	0.9998	0.08	0.26	3.3	5.1
Methoxychlor (MC)	0.30–250	0.9989	0.09	0.30	2.4	2.5

### 3.3. LOD, LOQ, and Precision

The lowest concentration or the lowest content of the target component that can be detected is considered as the detection limit (LOD). LOQ is the lowest concentration or the lowest quantity of the components to be measured in the sample by analytical method. The LOD and LOQ for each analytes were determined at the lowest concentration at a signal‐to‐noise ratio (S/N) of 3 and 10, respectively [28]. In this work, the calculated LOD and LOQ were tested experimentally. The LODs and LOQ for all targeted pesticides were determined using S/N three and 10 times, respectively, and were ranged from 0.05 to 0.88 and 0.16 to 2.93 μg/L, respectively. Table 1 shows that LOD (3 S/N) and LOQ (10 S/N) varied correspondingly from 0.05 to 0.88 μg/kg and from 0.16 to 2.93 μg/kg for all the analyzed compounds, respectively.

The precision of the method was determined by repeatability (intraday) and intermediate precision (interday). The intraday precision was determined as the relative standard deviation (%RSD) of results from triplicate measurements on the same day, and the interday precision was calculated as %RSD of results from three replicates performed within five consecutive days. The assay values for both variables were below 10% for all the analytes (Table 1).

### 3.4. Recovery Studies of the Method

For the recovery studies, 15.0 g of homogenously mixed and ground pesticide‐free samples were spiked with a mixture of selected pesticides at specified concentration levels. These samples were then kept at room temperature to facilitate the adsorption of pesticides onto them. A brief vibration using a vortex mixer was employed to ensure thorough dispersion of the solvent and the pesticides throughout the samples. To maintain matrix consistency in the final extract, fortification with larger volumes of the standard was minimized. Subsequently, the samples were processed following the previously specified method. The recoveries for all analytes were found to range between 97.70% and 115.90%, with RSDs remaining below 10%, as detailed in Table 2. These findings demonstrate that the chosen GC‐MS/MS method is precise, accurate, and sensitive enough for the quantitative analysis of the selected pesticides in sesame seed samples. The method meets the validation criteria, with all individual recoveries and RSD within the acceptable range outlined by the U.S. Department of Health and Human Services, Food and Drug Administration, specifically referenced in the Center for Drug Evaluation and Research (CDER) and Center for Veterinary Medicine (CVM) Guidance for Industry on Bioanalytical Method Validation (70% ≤ Q ≤ 120% and RSD ≤ 20%) for 20 OCPs in sesame seed samples [29]. The European Union has established specific regulations regarding the contamination levels in food products, including sesame seeds. These regulations are primarily aimed at ensuring food safety and protecting public health. The limits for contaminants such as aflatoxins, pesticides, and heavy metals are particularly important. The contamination levels and EU limits for Sesame seeds with OCPs using the AOAC 2007 method are presented in Table 3.

**Table 2:** Recovery ± %RSD results of all the analyzed pesticides.

Pesticides	Aldrin	α‐BHC	β‐BHC	*δ*‐BHC	Lindane	δ‐Chlordane	γ‐Chlordane	4,4′‐DDD	4,4′‐DDE	4,4′‐DDT	Dieldrin	α‐ES	β‐ES	ESS	Endrin	EA	EK	HC	HCE	MC
Blank reading (μg/L)	2.6 × 10^−3^	1.2 × 10^−3^	7.2 × 10^−3^	2.3 × 10^−3^	6.4 × 10^−3^	3.1 × 10^−3^	5.0 × 10^−4^	3.5 × 10^−2^	2.0 × 10^−3^	5.0 × 10^−3^	1.6 × 10^−2^	2.3 × 10^−2^	5.7 × 10^−3^	1.9 × 10^−2^	2.7 × 10^−2^	2.7 × 10^−2^	2.910^−2^	1.0 × 10^−3^	6.0 × 10^−4^	1.2 × 10^−3^
Spiked sample reading (20 μg/L)	18.718	19.56	20.62	19.66	19.87	19.70	20.18	23.65	20.86	21.91	21.91	23.18	21.26	21.87	20.16	20.23	20.30	20.25	21.70	21.35
Recovery ± RSD (%)	93.58	97.68	103.06	98.28	99.35	98.51	100.92	118.08	104.30	109.53	109.49	115.81	106.26	109.37	100.67	101.01	101.49	101.24	108.50	106.69

*Note: n* is the number of repeated measurement.

**Table 3:** Contamination levels and EU limits for sesame seeds with OCPs using the AOAC 2007 method.

Pesticides	Aldrin	α‐BHC	β‐BHC	δ‐BHC	Lindane	δ‐Chlordane	γ‐Chlordane	4,4′‐DDD	4,4′‐DDE	4,4′‐DDT	Dieldrin	α‐ES	β‐ES	ESS	Endrin	EA	EK	HC	HCE	MC
Study result (μg/L)	< LOD	0.001	0.001	0.001	< LOD	< LOD	< LOD	0.002	0.001	< LOD	< LOD	< LOD	< LOD	< LOD	< LOD	< LOD	< LOD	< LOD	< LOD	< LOD
EU, MRL (mg/kg)	0.01	0.05	0.05	0.05	0.01	0.01	0.05	0.05	0.05	0.01	0.05	0.01	0.01	0.01	0.01	0.01	0.01	0.01	0.01	0.01

Abbreviations: EA, endrin aldehyde; EK, endrin ketone; ES, endosulfan; ESS, endosulfan sulfate; HC, heptachlor; HCE, heptachlor epoxide; MC, methoxychlor.

### 3.5. Real Samples Analysis

The analysis of real sesame seed samples for 20 OCPs were conducted using a streamlined QuEChERS extraction and cleanup method, followed by detection via GC‐MS/MS in the MRM mode. Sesame seeds were selected due to their high oil content, which can complicate pesticide residue analysis by coextracting interfering compounds. The QuEChERS method effectively minimized matrix effects, ensuring accurate quantification and identification of OCPs at trace levels.

**Figure 2:**
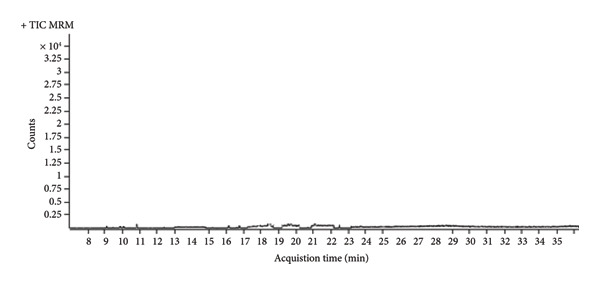
Real sample analysis chromatogram.

Prior to analysis, sesame seed samples were homogenized, and an acetonitrile‐based extraction was performed with salts (MgSO_4_ and NaCl) to induce phase separation. A d‐SPE cleanup step using PSA and C18 sorbents was applied to remove fatty acids and other coextractives. The purified extracts were then analyzed by GC‐MS/MS in the MRM mode, which enhanced selectivity and sensitivity by monitoring specific precursor‐to‐product ion transitions for each OCP (Figure 2).

Method validation confirmed excellent linearity (*R* ^2^ ≥ 0.999), precision (RSD < 10%), and recoveries (90%–115%) across blank‐spiked concentrations. The LODs and LOQs were in the ranges of 0.05–0.88 μg/kg and from 0.16 to 2.93 μg/kg, respectively, complying with MRLs set by regulatory agencies. Real sample screening revealed trace levels of certain OCPs, demonstrating the method’s effectiveness in routine monitoring and food safety compliance. The combination of QuEChERS and GC‐MS/MS in the MRM mode proved to be a robust, high‐throughput approach for multiresidue pesticide analysis in complex matrices like sesame seeds.

As shown in the chromatogram in Figure 2, there were no interfering peaks near the acquisition time of analytes, thus allowing the analyte to be quantified. That is, no detectable pesticides in the collected sesame seeds.

The individual GC‐MS/MS chromatograms of all analytes are given in Supporting Information (SI 2a‐t), and the scan results were evaluated using Agilent enhanced mass transfer GC/MS data Acquisition work station software. Ions are separated in the first quadrupole, fragmented further in a collision cell, and the product ions are separated and analyzed by a second quadrupole as shown in Table 4. Triple quadruple GC‐MS did not suffer from interferences nor does require dual columns to confirm the identity of peaks. Target analytes were identified by monitoring two transition ions where possible. The most dominant transition ion was used for quantification whereas the second most intense ion as a qualifier for confirmation purposes. The use of precursor and product ions in mass spectral analysis allowed for sensitive detection and confirmation of all pesticides in this sample.

**Table 4:** The quantitative analysis data of 20 pesticides.

Compound name	RT (min)	Quantitation MRM (CE)	Qualifier MRM 1 (CE)	Qualifier MRM 2 (CE)
α‐BHC	20.55	180.9 ⟶ 145 (15)	217 ⟶ 181 (5)	219 ⟶ 183 (5)
Lindane	21.28	180.9 ⟶ 145 (15)	217 ⟶ 181 (5)	219 ⟶ 183 (5)
β‐BHC	21.42	180.9 ⟶ 145 (15)	217 ⟶ 181 (5)	219 ⟶ 183 (5)
δ‐BHC	22.06	180.9 ⟶ 145 (15)	217 ⟶ 181 (5)	219 ⟶ 183 (5)
Heptachlor	23.33	272 ⟶ 236.9 (15)	236.8 ⟶ 118.9 (25)	273.9 ⟶ 238.9 (15)
Aldrin	24.09	263 ⟶ 193 (35)	263 ⟶ 191 (35)	263 ⟶ 263 (0)
Heptachlor epoxide	25.07	272 ⟶ 236.9 (15)	262.9 ⟶ 192.9 (35)	273.9 ⟶ 238.9 (15)
γ‐Chlordane	25.63	272 ⟶ 236.9 (15)	273.9 ⟶ 238.9 (15)	277 ⟶ 241 (5)
α‐Endosulfan	25.91	195.1 ⟶ 159 (5)	272 ⟶ 236.9 (15)	273.9 ⟶ 238.9 (15)
α‐Chlordane	26.00	272 ⟶ 236.9 (15)	273.9 ⟶ 238.9 (15)	277 ⟶ 241 (5)
4,4′‐DDE	26.53	246.1 ⟶ 176.2 (30)	315.8 ⟶ 246 (15)	317.8 ⟶ 317.8 (0)
Dieldrin	26.54	263 ⟶ 191 (35)	277 ⟶ 241 (5)	263 ⟶ 193 (35)
Endrin	27.03	263 ⟶ 193 (35)	263 ⟶ 191 (35)	263 ⟶ 263 (0)
β‐Endosulfan	27.25	195.1 ⟶ 159 (5)	206.9 ⟶ 172 (15)	180.9 ⟶ 145 (15)
4,4′‐DDD	27.48	235.1 ⟶ 165.2 (20)	237 ⟶ 165.1 (27)	237 ⟶ 237 (0)
Endrin aldehyde	27.70	184.9 ⟶ 121 (15)	206.9 ⟶ 172 (15)	195.1 ⟶ 159 (5)
Endosulfan sulfate	28.23	272 ⟶ 236.9 (15)	273.9 ⟶ 238.9 (15)	277 ⟶ 241 (5)
4,4′‐DDT	28.33	235.1 ⟶ 165.2 (20)	237 ⟶ 237 (0)	237 ⟶ 165.1 (27)
Endrin ketone	29.27	180.9 ⟶ 145 (15)	114.9 ⟶ 51.1 (25)	195.1 ⟶ 159 (5)
Methoxychlor	29.64	227 ⟶ 141.1 (40)	227 ⟶ 169.1 (30)	227 ⟶ 212 (17)

Abbreviation: CE, collision energy.

## 4. Comparison of Results With Previously Published Methods

Table 5 demonstrates measurable improvements in LOD/LOQ and recovery percentages for the analyzed sample compared with the other reported methods. The result was compared with other methods for different classes of pesticides considering analytical characteristics including LOD, LOD, RSD, and percent recovery (Table 5). It can be noted that the results of this proposed method are comparable with the other reported methods either for OCPs or other reported pesticides. Moreover, LODs, (%) RSD, and (%) recovery values are better than those reported in the other papers. The MS/MS detection system that shows very low LODs in the present work is more sensitive than FID or ECD as shown on the other reports. Therefore, the QuEChERS–GC‐MS/MS method can be a good option as a simple, cheap, green, and available technique for the analysis of the selected pesticides.

**Table 5:** Comparison of the results of this work with previously published methods.

Matrices	Analytes	LOD (μg/kg)	LOQ (μg/kg)	RSD (%)	Recoveries (%)	Method of detection	Reference
Sesame	OCPs	1	5–10	**< 12**	84–102	GC‐MS	[30]

Spices	OCPs and synthetic pyrethroid				75–90%	GC–MS	

Triton *X*‐100	OCPs and OPPs	0.03–0.26	0.100.88	1–4	72–105 5–85	GC‐MS and GC‐MS/MS	[31]

Biological samples	PCBs, PAHs, PBDEs and PCDD/Fs	0.05–1		5–15	70	Gel Permeation Chromatography (GPC)	[32]

Sesame seeds	12 OCPs	1.6–3.3	5–10	< 20	> 75	GC‐ECD	[33]

Oil seed including sesame	Multiple pesticide residues	6–54		< 15	80.2–99.8	Sin‐QuEChERS LC‐MS/MS	[27]

Edible oil samples	229 pesticide	0.9–2.8	3.0–9.2	3.3–5.6	68–127	QuEChERS‐GC‐MS/MS	[34]
		0.300–0.335	0.990–1.102	2.6–3.0	92–102	SPME‐MMIPF‐GC‐MS	

White sesame seed	20 OCPs	0.05–0.88	0.16–2.93	< 10	90–115	QuEChERS‐GC‐MS	This work

## 5. Conclusion

The application of GC‐MS/MS in the MRM mode for the rapid detection of 20 OCPs in sesame seeds demonstrates a significant advancement in analytical chemistry and food safety monitoring. The use of QuEChERS sample cleanup method enhances the efficiency and effectiveness of the extraction process, allowing for the successful isolation of OCPs from sesame seeds. The method provides several advantages, including reduced solvent usage, shorter preparation times, and the ability to handle multiple samples simultaneously. This is particularly beneficial for routine analysis in laboratories where throughput and efficiency are critical. The combination of QuEChERS with GC‐MS/MS in the MRM mode allows for high specificity and sensitivity in detecting low concentrations of OCPs, which is essentially given the potential health risks associated with pesticide residues in food products. The use of MRM mode enhances the analytical performance by enabling the simultaneous quantification of multiple target compounds with minimal interference from coeluting substances. This capability is crucial for ensuring compliance with regulatory limits and safeguarding consumer health. The method’s robustness and reliability further underscore its applicability in routine monitoring programs. Finally, this study highlights the effectiveness of integrating QuEChERS sample preparation with GC‐MS/MS in the MRM mode for the rapid and accurate detection of OCPs in sesame seeds. It paves the way for future research and developments in food safety testing methodologies, ultimately contributing to better monitoring practices and improved public health outcomes. Further studies could explore the extension of this methodology to other food matrices and a broader range of contaminants, thereby enhancing food safety surveillance systems globally.

## Ethics Statement

This article does not contain any studies with human or animal subjects.

## Consent

The authors have nothing to report.

## Disclosure

I/we affirm that the research was conducted independently and that the findings presented in this manuscript are solely those of the authors.

## Conflicts of Interest

In accordance with the guidelines of the *Journal of Analytical Methods in Chemistry*, I/we hereby declare that there are no conflicts of interest associated with this manuscript titled “Enhanced Detection of Organochlorine Pesticide Residues in Sesame Seeds (*Sesamum indicum L*.) Using Advanced GC‐MS/MS Techniques.” We have disclosed any financial, personal, or professional relationships that could be perceived as potential conflicts of interest.

## Funding

No fund was received for this study.

## Supporting Information

Additional supporting information can be found online in the Supporting Information section.

## Supporting information


**Supporting Information 1** Supporting Information A.

**Supporting Information 2** Supporting Information B.

## Data Availability

The data that support the findings of this study are available in the supporting information of this article.

## References

[1] Wei P. , Zhao F. , Wang Z. et al., Sesame (*Sesamum indicum L.*): A Comprehensive Review of Nutritional Value, Phytochemical Composition, Health Benefits, Development of Food, and Industrial Applications, Nutrients. (2022) 14, no. 19, 10.3390/nu14194079. PMC9573514 36235731

[2] Teklu D. H. , Shimelis H. , Tesfaye A. , and Abady S. , Appraisal of the Sesame Production Opportunities and Constraints, and Farmer-Preferred Varieties and Traits, in Eastern and Southwestern Ethiopia, Sustainability. (2021) 13, no. 20, 10.3390/su132011202.

[3] Negatu B. , Dugassa S. , and Mekonnen Y. , Environmental and Health Risks of Pesticide Use in Ethiopia, Journal of Health and Pollution. (2021) 11, no. 30, 10.5696/2156-9614-11.30.210601. PMC8276724 34267988

[4] Ajiboye T. O. , Kuvarega A. T. , and Onwudiwe D. , Recent Strategies for Environmental Remediation of Organochlorine Pesticides, Applied Sciences. (2020) 10, no. 18, 10.3390/app10186286.

[5] Chandra R. , Sharpanabharathi N. , Prusty B. A. K. , Azeez P. A. , and Kurakalva R. M. , Organochlorine Pesticide Residues in Plants and Their Possible Ecotoxicological and Agri Food Impacts, Scientific Reports. (2021) 11, no. 1, 10.1038/s41598-021-97286-4. PMC8426456 34497319

[6] Zaynab M. , Fatima M. , Sharif Y. et al., Health and Environmental Effects of Silent Killers Organochlorine Pesticides and Polychlorinated Biphenyl, Journal of King Saud University Science. (2021) 33, no. 6, 10.1016/j.jksus.2021.101511.

[7] Mekonnen E. , Assessing the Core Challenges on the Process of Exporting Quality Sesame Seeds in Ethiopia, 2019, St. Mary’s University, Addis Ababa, Ethiopia, Doctoral Dissertation.

[8] Angamuthu M. , Kasirajan S. , Pandurangan G. , Langyan G. , and Perumal V. , Langyan S. , Yadava P. , Yadav R. , and Bhardwaj R. , Organic Production of Sesame: Status, Techniques, and Market, Sesame: Sustainable Production and Applications, 2025, Springer, Berlin, Germany, 81–105.

[9] Bhawan F. , Guidance Document and Standard Operating Procedures for Fixation of Maximum Residue Limits (MRLs) of Pesticides in Food Commodities, 2022, Food Safety & Standards Authority of India (FSSAI).

[10] Wahab S. , Muzammil K. , Nasir N. et al., Advancement and New Trends in Analysis of Pesticide Residues in Food: A Comprehensive Review, Plants. (2022) 11, no. 9, 10.3390/plants11091106. PMC9105315 35567107

[11] Ye X. S. , Shao H. , Zhou T. , Xu J. , Cao X. , and Mo W. , Analysis of Organochlorine Pesticides in Tomatoes Using a Modified QuEChERS Method Based on N-Doped Graphitized Carbon Coupled With GC-MS/MS, Food Analytical Methods. (2020) 13, no. 3, 823–832, 10.1007/s12161-019-01674-6.

[12] Stalikas C.D. , Extraction, Separation, and Detection Methods for Phenolic Acids and Flavonoids, Journal of Separation Science. (2007) 30, no. 18, 3268–3295, 10.1002/jssc.200700261, 2-s2.0-37849002920. 18069740

[13] de Freitas L. V. P. , Alves L. M. G. , Sicupira L. C. , de Pinho G. P. , and Silvério F. O. , Determination of DDT in Honey Samples by Liquid–Liquid Extraction With Low-Temperature Purification (LLE-LTP) Combined to HPLC-DAD, Analytical Methods. (2021) 13, no. 16, 1955–1964, 10.1039/d1ay00264c. 33913942

[14] Sharif Z. , Man Y. B. C. , Hamid N. S. A. , and Keat C. C. , Determination of Organochlorine and Pyrethroid Pesticides in Fruit and Vegetables Using Solid Phase Extraction Clean-Up Cartridges, Journal of Chromatography A. (2006) 1127, no. 1-2, 254–261, 10.1016/j.chroma.2006.06.007, 2-s2.0-33748764149. 16857206

[15] Dabbagh M. S. and Farajzadeh M. A. , Introduction of a New Procedure for the Synthesis of Polysulfone Magnetic Nanoparticles and Their Application in Magnetic Solid Phase Extraction for the Extraction of Some Pesticides From Fruit and Vegetable Juices, Microchemical Journal. (2020) 158, 10.1016/j.microc.2020.105238.

[16] Kumar S. , Kumar R. , and Kumar D. , Advanced Strategies for Enhancing Analytical Techniques of Solid Phase Microextraction: An Overview, Current Green Chemistry. (2025) 12, 10.2174/0122133461384287250711063801.

[17] Perestrelo R. , Silva P. , Porto-Figueira P. et al., QuEChERS-Fundamentals, Relevant Improvements, Applications and Future Trends, Analytica Chimica Acta. (2019) 1070, 1–2, 10.1016/j.aca.2019.02.036, 2-s2.0-85062648582. 31103162

[18] Montiel-León J. M. , Duy S. V. , Munoz G. et al., Occurrence of Pesticides in Fruits and Vegetables From Organic and Conventional Agriculture by QuEChERS Extraction Liquid Chromatography Tandem Mass Spectrometry, Food Control. (2019) 104, 74–82, 10.1016/j.foodcont.2019.04.027, 2-s2.0-85064908749.

[19] Cabrera L. C. , Caldas S. S. , Prestes O. D. , Primel E. G. , and Zanella R. , Evaluation of Alternative Sorbents for Dispersive Solid‐Phase Extraction Clean-Up in the QuEChERS Method for the Determination of Pesticide Residues in Rice by Liquid Chromatography With Tandem Mass Spectrometry, Journal of Separation Science. (2016) 39, no. 10, 1945–1954, 10.1002/jssc.201501204, 2-s2.0-84992300380. 27004927

[20] Correia-Sá L. , Fernandes V. C. , Calhau C. , Domingues V. F. , and Delerue-Matos C. , Optimization of QuEChERS Procedure Coupled to GC-ECD for Organochlorine Pesticide Determination in Carrot Samples, Food Analytical Methods. (2013) 6, no. 2, 587–597, 10.1007/s12161-012-9463-x, 2-s2.0-84875053315.

[21] Łozowicka B. , Rutkowska E. , and Jankowska M. , Influence of QuEChERS Modifications on Recovery and Matrix Effect During the Multi-Residue Pesticide Analysis in Soil by GC/MS/MS and GC/ECD/NPD, Environmental Science and Pollution Research. (2017) 24, no. 8, 7124–7138, 10.1007/s11356-016-8334-1, 2-s2.0-85009433368. 28093672 PMC5383684

[22] Tankiewicz M. , Determination of Selected Priority Pesticides in High Water Fruits and Vegetables by Modified QuEChERS and GC-ECD With GC-MS/MS Confirmation, Molecules. (2019) 24, no. 3, 10.3390/molecules24030417, 2-s2.0-85060535913. PMC6384567 30678356

[23] Papadakis E. N. , Vryzas Z. , and Papadopoulou-Mourkidou E. , Rapid Method for the Determination of 16 Organochlorine Pesticides in Sesame Seeds by Microwave-Assisted Extraction and Analysis of Extracts by Gas Chromatography–Mass Spectrometry, Journal of Chromatography A. (2006) 1127, no. 1-2, 6–11, 10.1016/j.chroma.2006.06.010, 2-s2.0-33747800055. 16797565

[24] Aysheshm K. , Sesame Market Chain Analysis: The Case of Metema Woreda, North Gondar Zone, Amhara National Regional State, 2007, Haramaya University, Haramaya, Ethiopia, Doctoral Dissertation.

[25] Shinde R. , Pardeshi A. , Dhanshetty M. , Anastassiades M. , and Banerjee K. , Development and Validation of an Analytical Method for the Multiresidue Analysis of Pesticides in Sesame Seeds Using Liquid-and Gas Chromatography With Tandem Mass Spectrometry, Journal of Chromatography A. (2021) 1652, 10.1016/j.chroma.2021.462346. 34186324

[26] Galmiche M. , Rodrigues A. , Motsch E. , Delhomme O. , François Y. , and Millet M. , The Use of Pseudo‐Mrm for a Sensitive and Selective Detection and Quantification of Polycyclic Aromatic Compounds by Tandem Mass Spectrometry, Rapid Communications in Mass Spectrometry. (2022) 36, no. 13, 10.1002/rcm.9307. 35355348

[27] Hua J. , Fayyaz A. , Song H. , Tufail M. R. , and Gai Y. , Development of a Method Sin-QuEChERS for the Determination of Multiple Pesticide Residues in Oilseed Samples, Quality Assurance and Safety of Crops and Foods. (2019) 11, no. 6, 511–516, 10.3920/qas2019.1557.

[28] Chen J. N. , Lian Y. J. , Zhou Y. R. et al., Determination of 107 Pesticide Residues in Wolfberry With Acetate-Buffered Salt Extraction and Sin-QuEChERS Nano Column Purification Coupled With Ultra Performance Liquid Chromatography Tandem Mass Spectrometry, Molecules. (2019) 24, no. 16, 10.3390/molecules24162918, 2-s2.0-85070680276. PMC6719108 31408943

[29] Smith G. , Bioanalytical Method Validation: Notable Points in the 2009 Draft EMA Guideline and Differences With the 2001 FDA Guidance, Bioanalysis. (2010) 2, no. 5, 929–935, 10.4155/bio.10.42, 2-s2.0-79952019611. 21083222

[30] Papadakis E. N. , Vryzas Z. , and Papadopoulou-Mourkidou E. , Rapid Method for the Determination of 16 Organochlorine Pesticides in Sesame Seeds by Microwave-Assisted Extraction and Analysisof Extracts by Gas chromatography–Mass Spectrometry, Journal of Chromatography A. (2006) 1127, no. 1-2, 6–11, 10.1016/j.chroma.2006.06.010, 2-s2.0-33747800055. 16797565

[31] Hristozova A. , Simitchiev K. K. , Kmetov V. , and Rosenberg E. , Compatibility of Cloud Point Extraction With Gas Chromatography: Matrix Effects of Triton X-100 on GC-MS and GC-MS/MS Analysis of Organochlorine and Organophosphorus Pesticides, Talanta. (2024) 269, 10.1016/j.talanta.2023.125445. 38039676

[32] Cloutier P. L. , Fortin F. , Groleau P. E. , Brousseau P. , Fournier M. , and Desrosiers M. , QuEChERS Extraction for Multi-Residue Analysis of PCBs, PAHs, PBDEs and PCDD/Fs in Biological Samples, Talanta. (2017) 165, 332–338, 10.1016/j.talanta.2016.12.080, 2-s2.0-85007508303. 28153263

[33] Wang G. Q. , Zhang D. F. , Wang S. F. , Sun Y. A. , and Sun X. L. , Determination of Organochlorine Pesticide Residues in Sesame Seeds by GC-ECD, Chroma. (2009) 69, no. 11-12, 1347–1351, 10.1365/s10337-009-1059-2, 2-s2.0-67649989678.

[34] Kardani F. , Jelyani A. Z. , Khezeli T. et al., Determination of 229 Pesticides Residue in Edible Oil Samples Using Conventional Quechers Method and Solid Phase Microextraction Based on Monolithic Molecularly Imprinted Polymer Fiber and Analysis With GC-MS, BioMed Central Chemistry. (2025) 19, no. 1, 10.1186/s13065-025-01518-x. PMC12102980 40413555

