# Supplementary material for: Enhanced Detection of Organochlorine Pesticide Residues in Sesame Seeds (Sesamum indicum L.) Using Advanced GC‐MS/MS Techniques
Source: J Anal Methods Chem. 2026 Jan 20;2026:8312847. doi: 10.1155/jamc/8312847 (PMC12817196; doi:10.1155/jamc/8312847)
Supplement: Supplementary file 1 — Supporting Information 1 Supporting Information A. [file JAMC-2026-8312847-s002.pptx]

## Slide 1
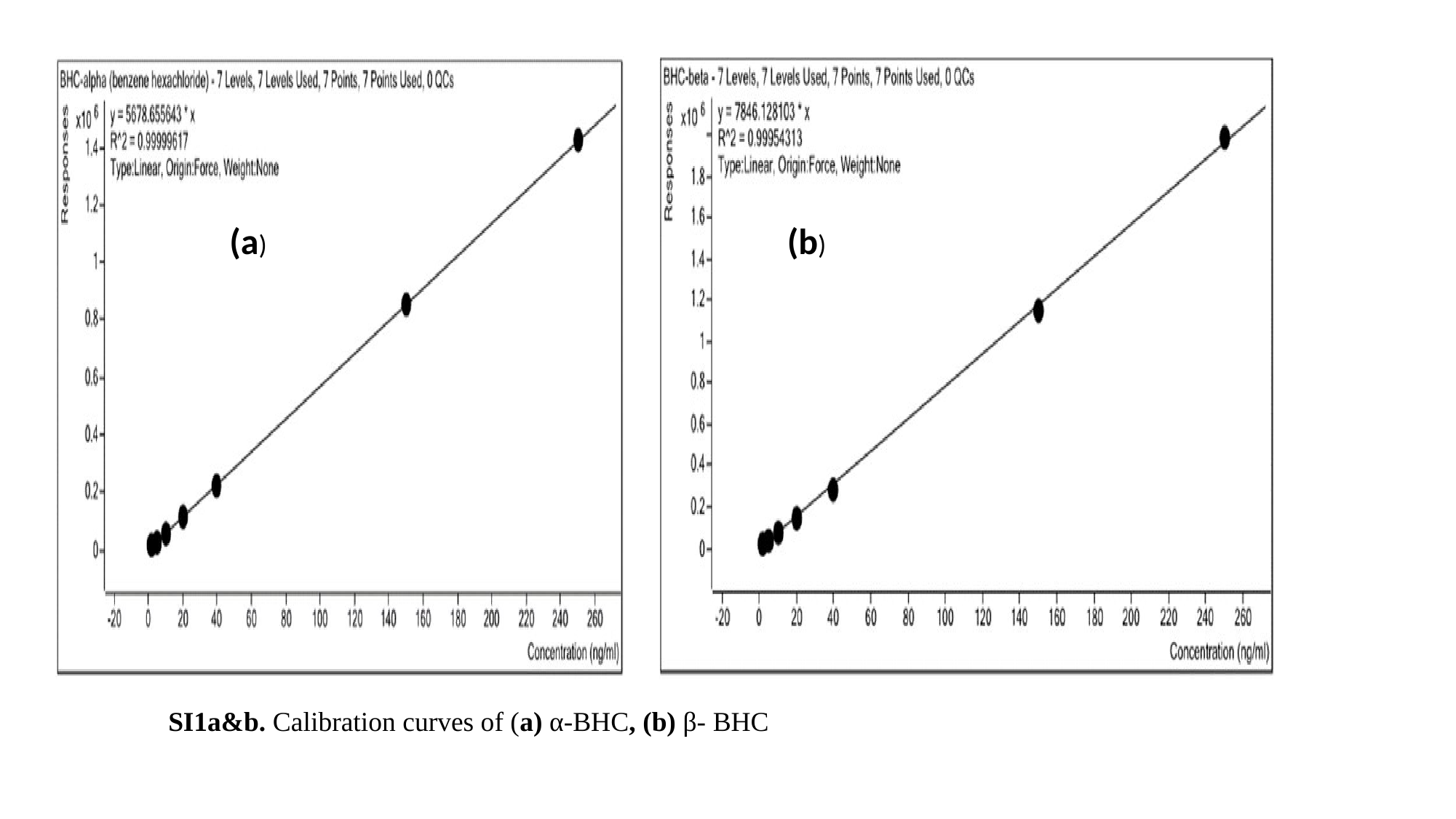

(b)
(a)
SI1a&b. Calibration curves of (a) α-BHC, (b) β- BHC

## Slide 2
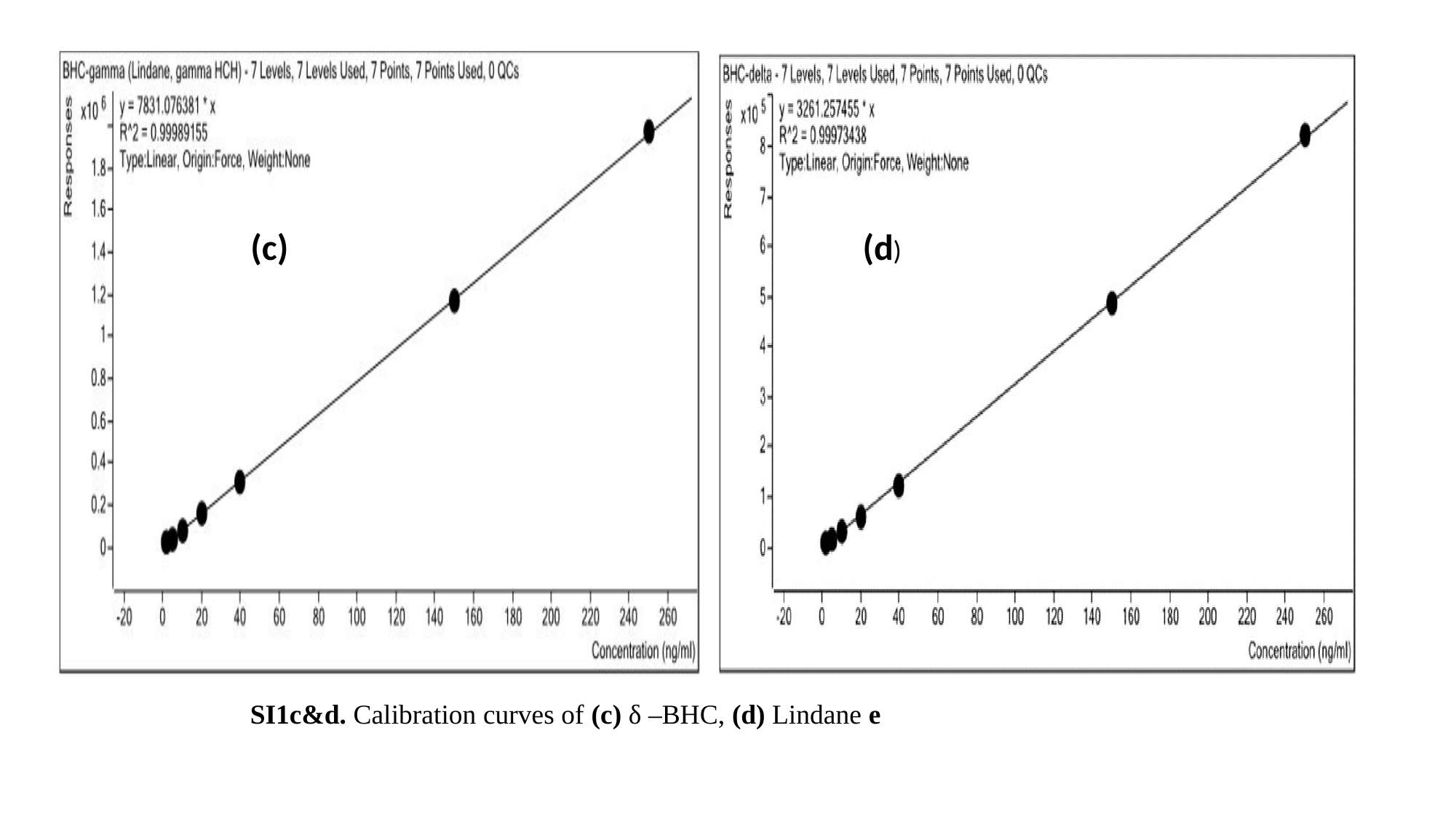

(c)
(d)
SI1c&d. Calibration curves of (c) δ –BHC, (d) Lindane e

## Slide 3
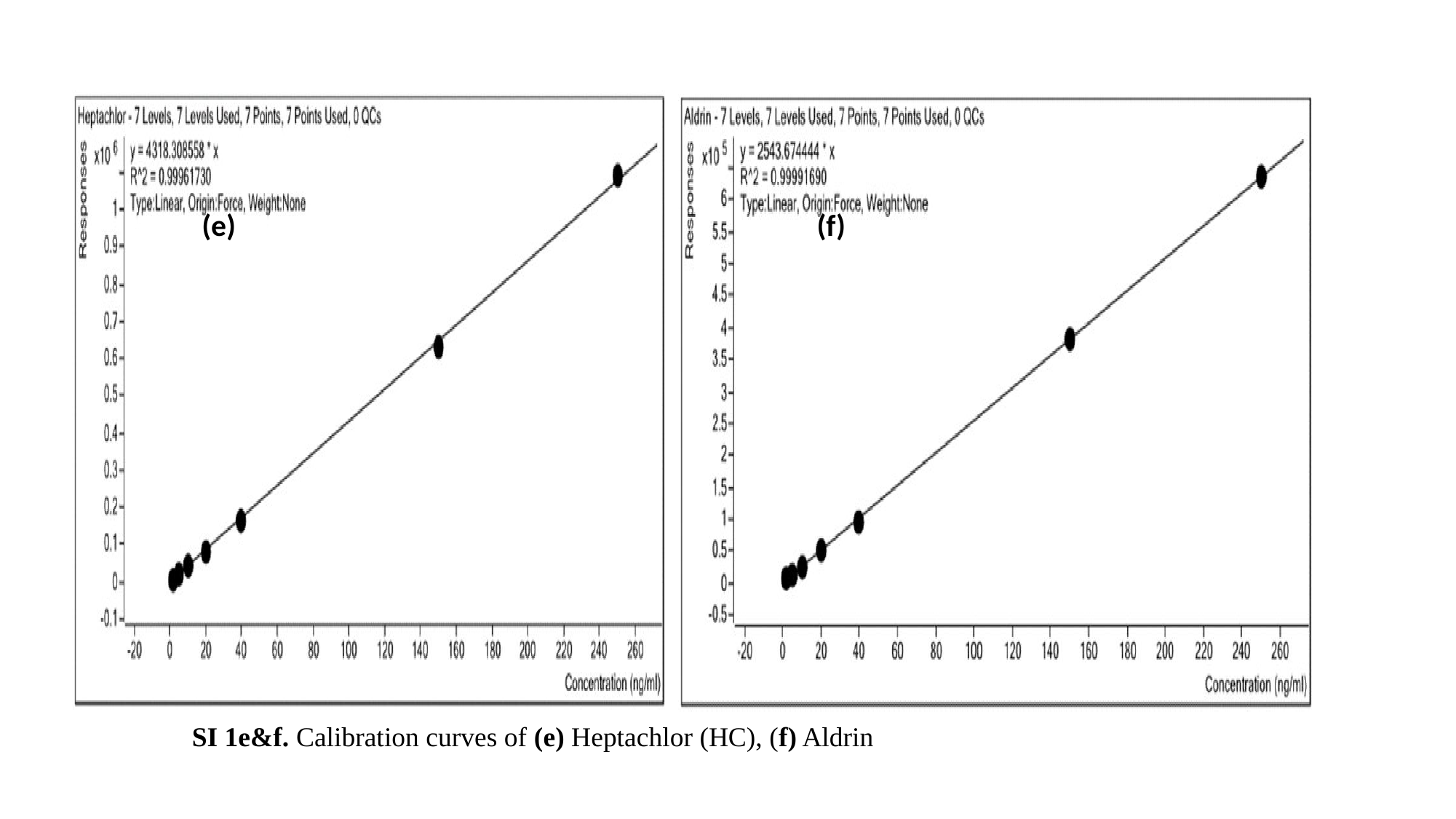

(e)
(f)
SI 1e&f. Calibration curves of (e) Heptachlor (HC), (f) Aldrin

## Slide 4
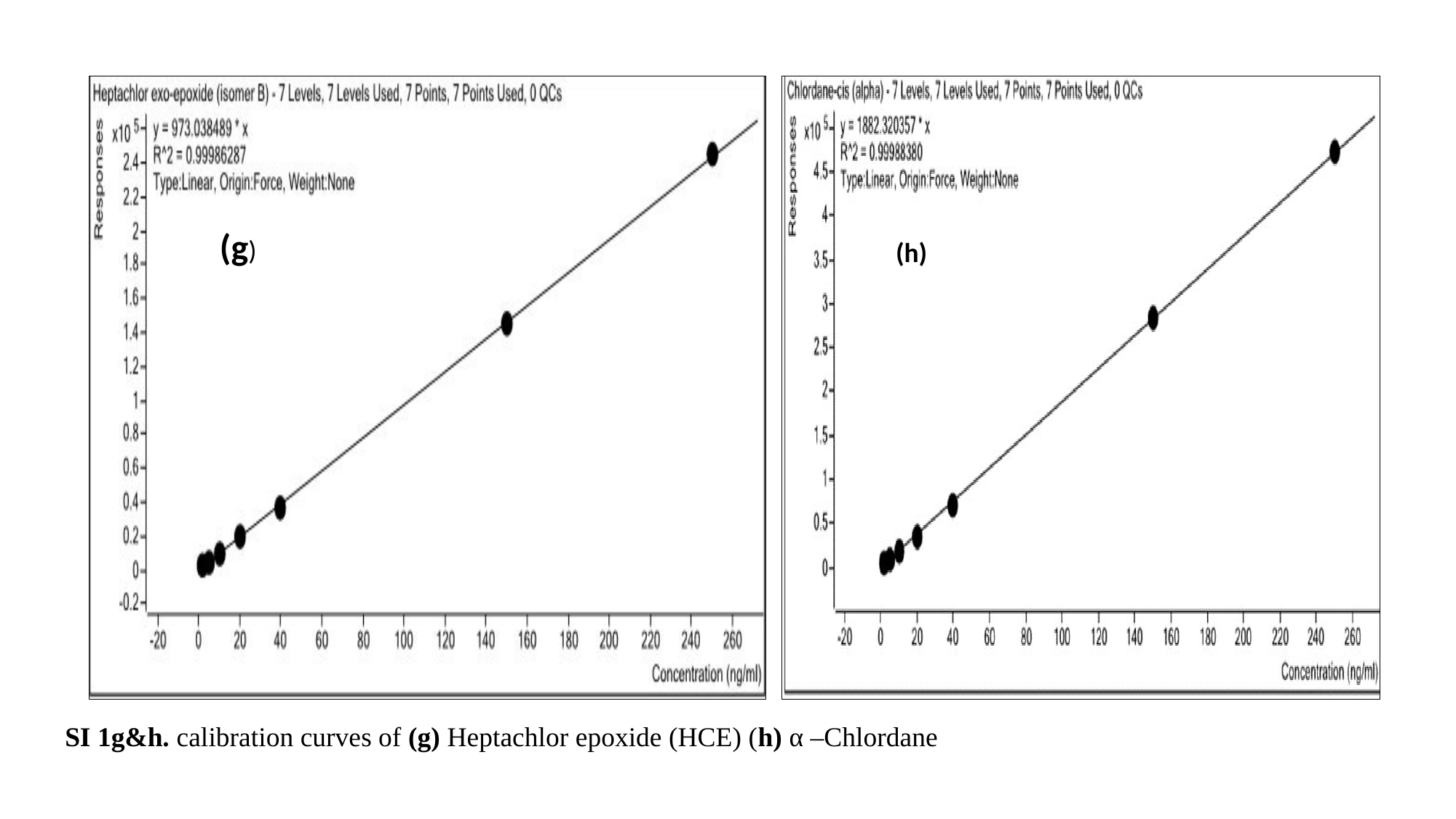

(g)
(h)
SI 1g&h. calibration curves of (g) Heptachlor epoxide (HCE) (h) α –Chlordane

## Slide 5
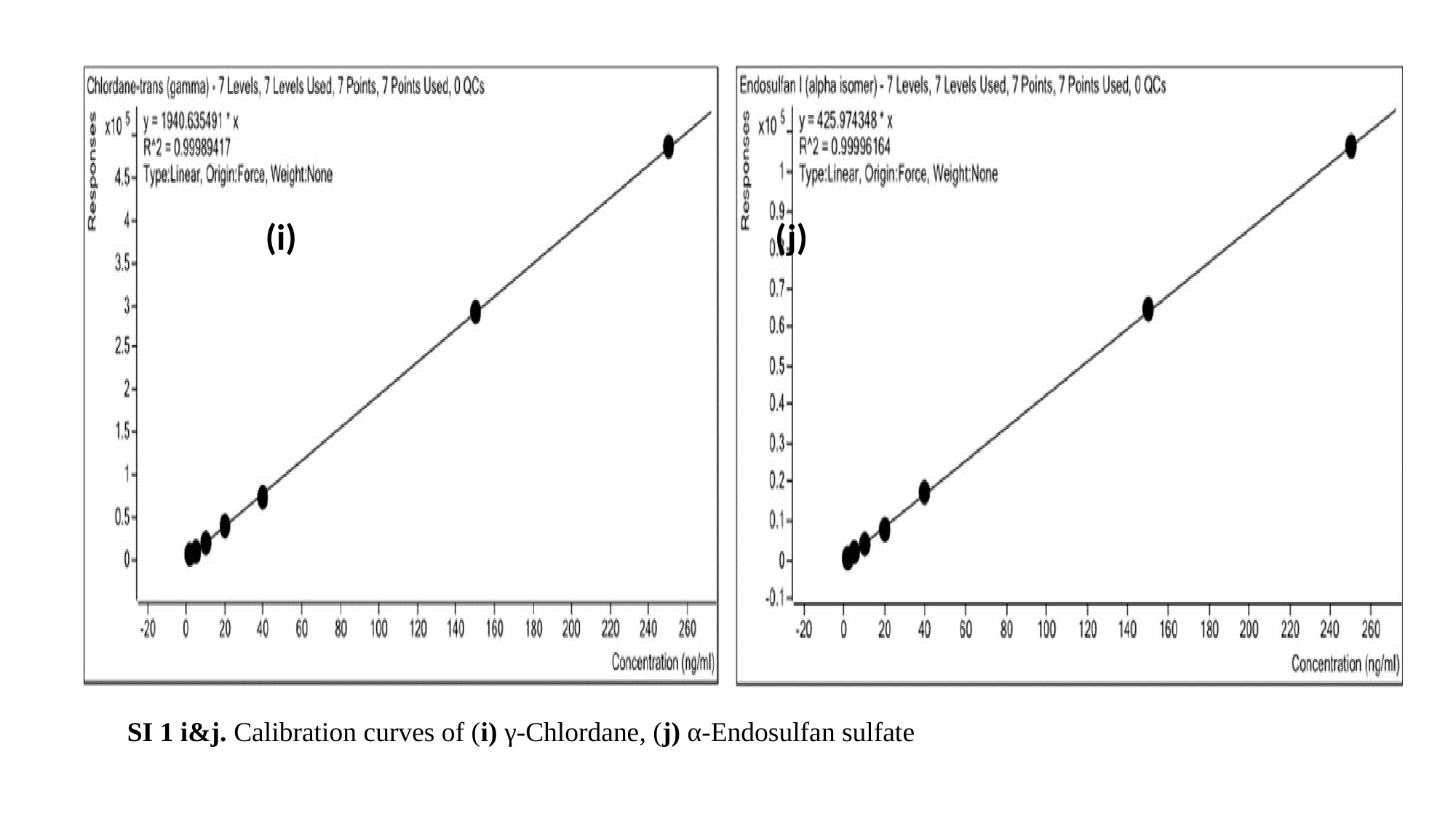

(j)
(i)
SI 1 i&j. Calibration curves of (i) γ-Chlordane, (j) α-Endosulfan sulfate

## Slide 6
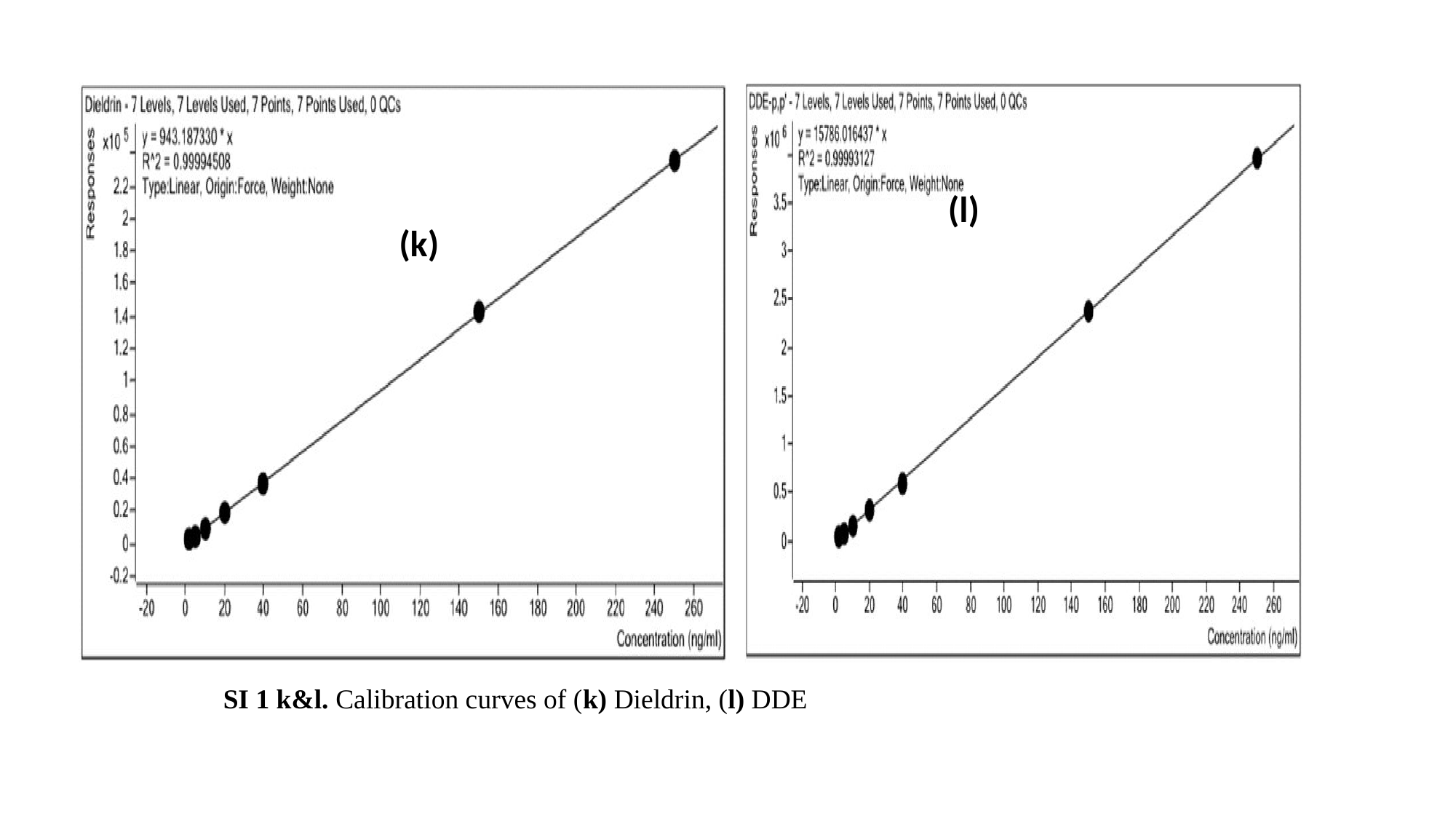

(l)
(k)
SI 1 k&l. Calibration curves of (k) Dieldrin, (l) DDE

## Slide 7
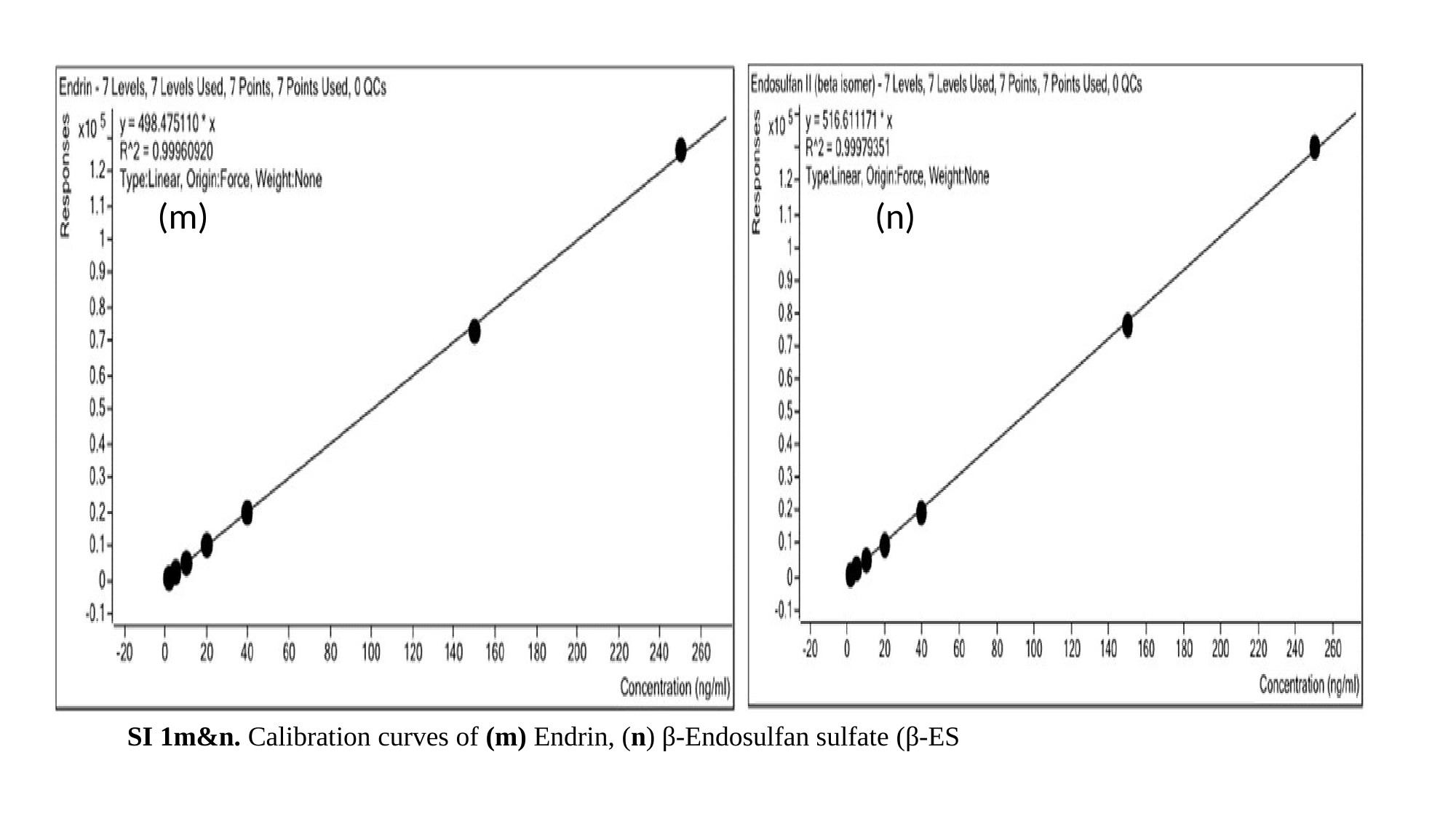

(m)
(n)
SI 1m&n. Calibration curves of (m) Endrin, (n) β-Endosulfan sulfate (β-ES

## Slide 8
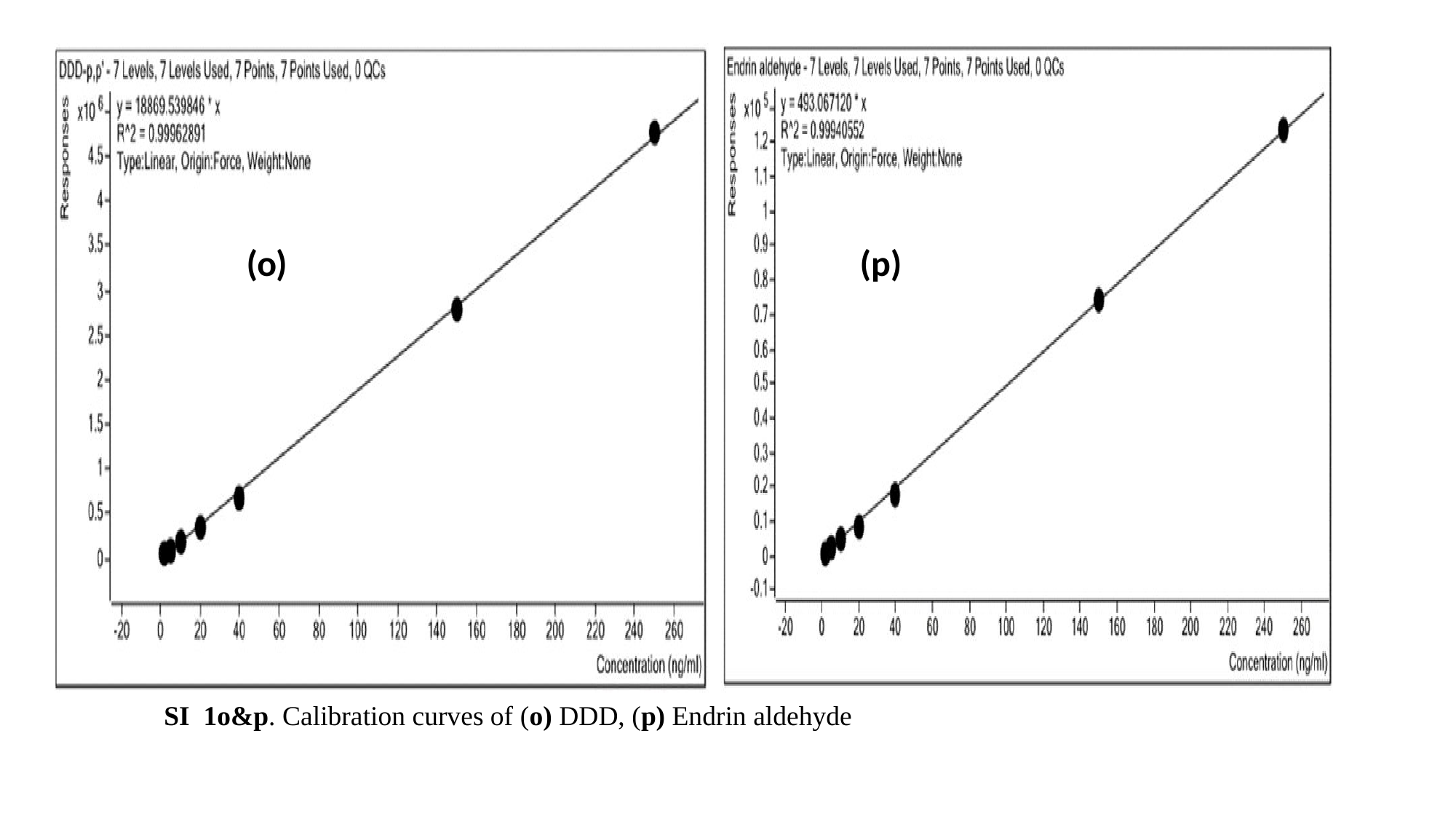

(p)
(o)
SI 1o&p. Calibration curves of (o) DDD, (p) Endrin aldehyde

## Slide 9
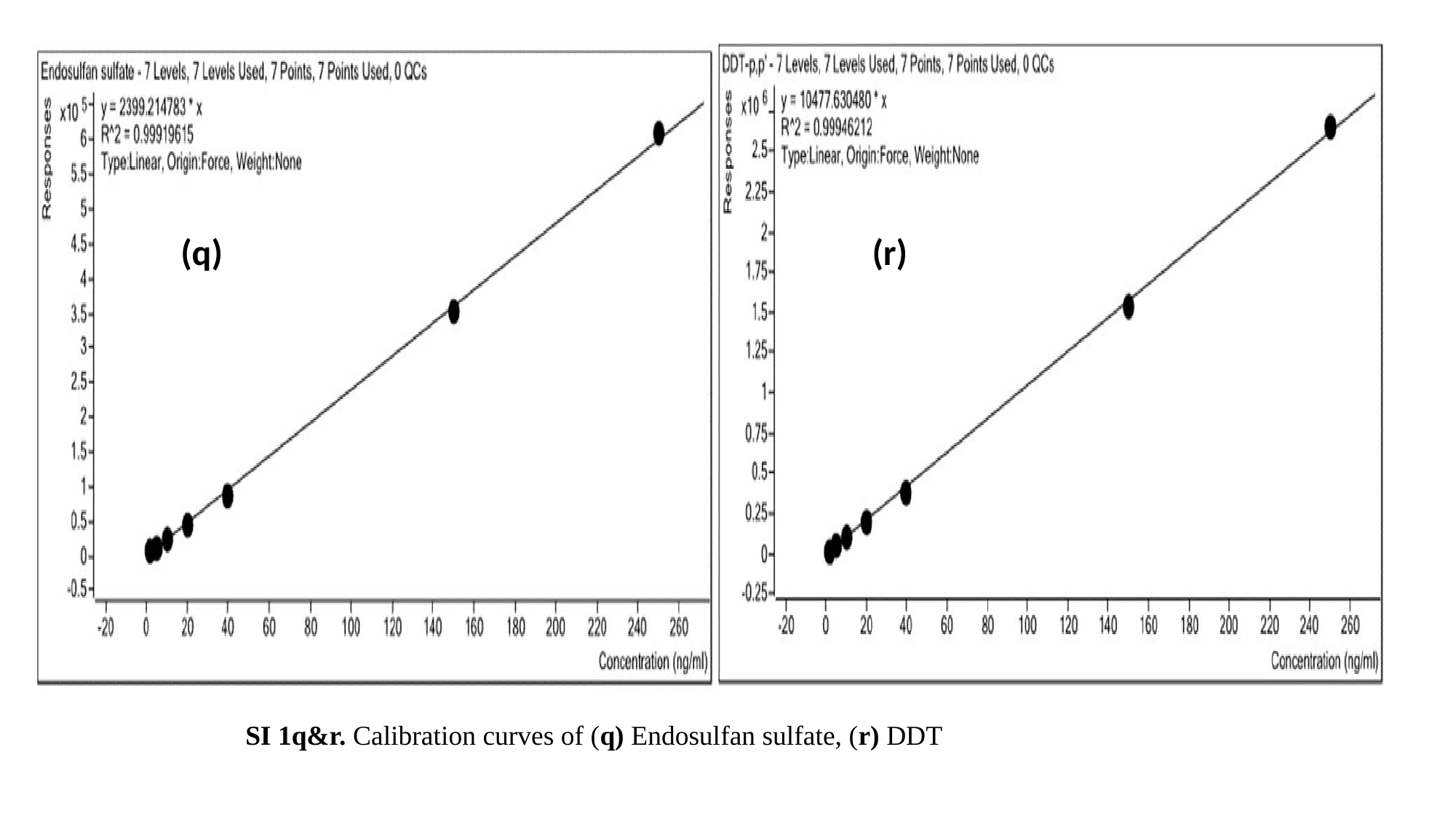

(q)
(r)
SI 1q&r. Calibration curves of (q) Endosulfan sulfate, (r) DDT

## Slide 10
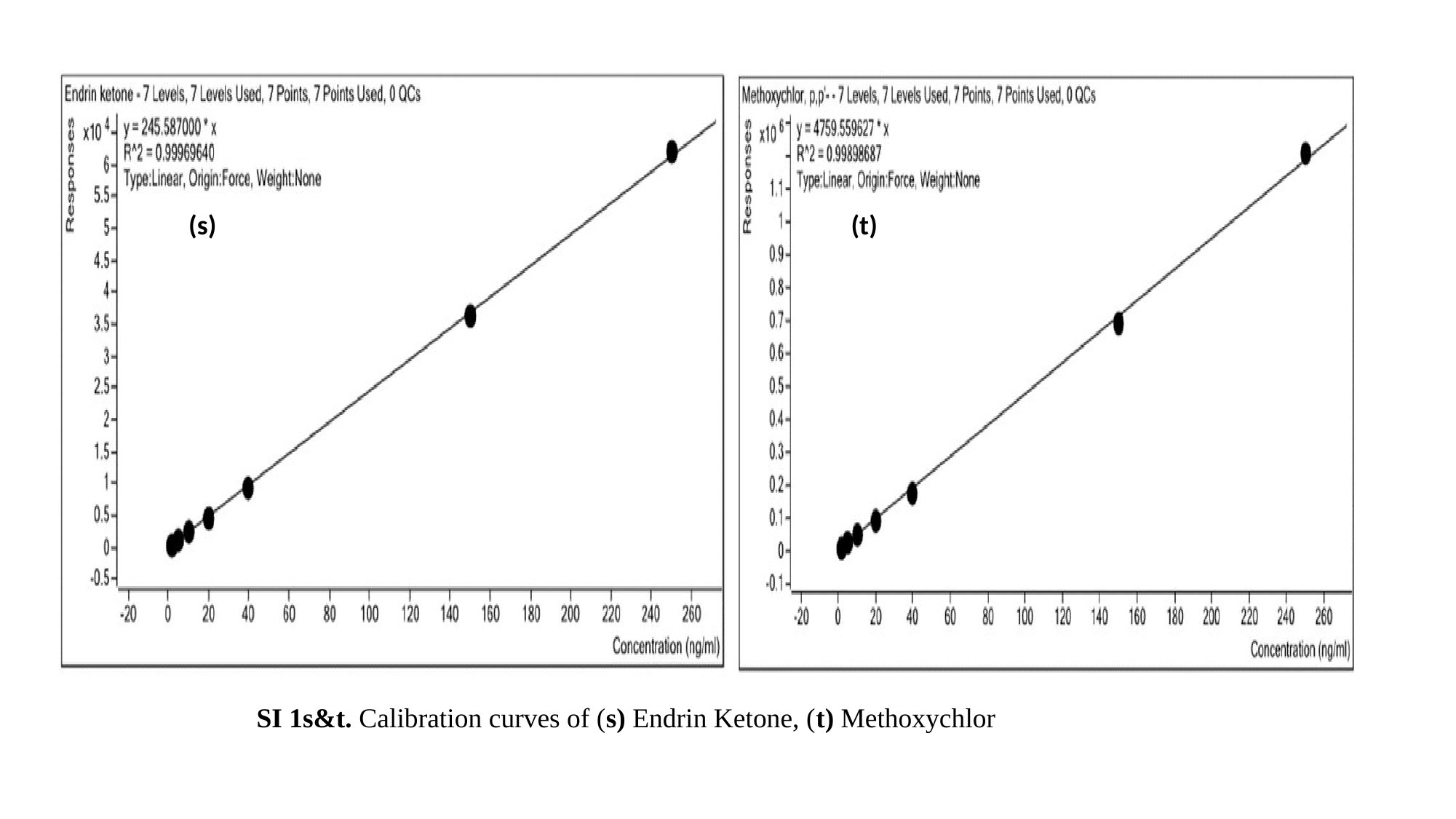

(s)
(t)
SI 1s&t. Calibration curves of (s) Endrin Ketone, (t) Methoxychlor

## Slide 11
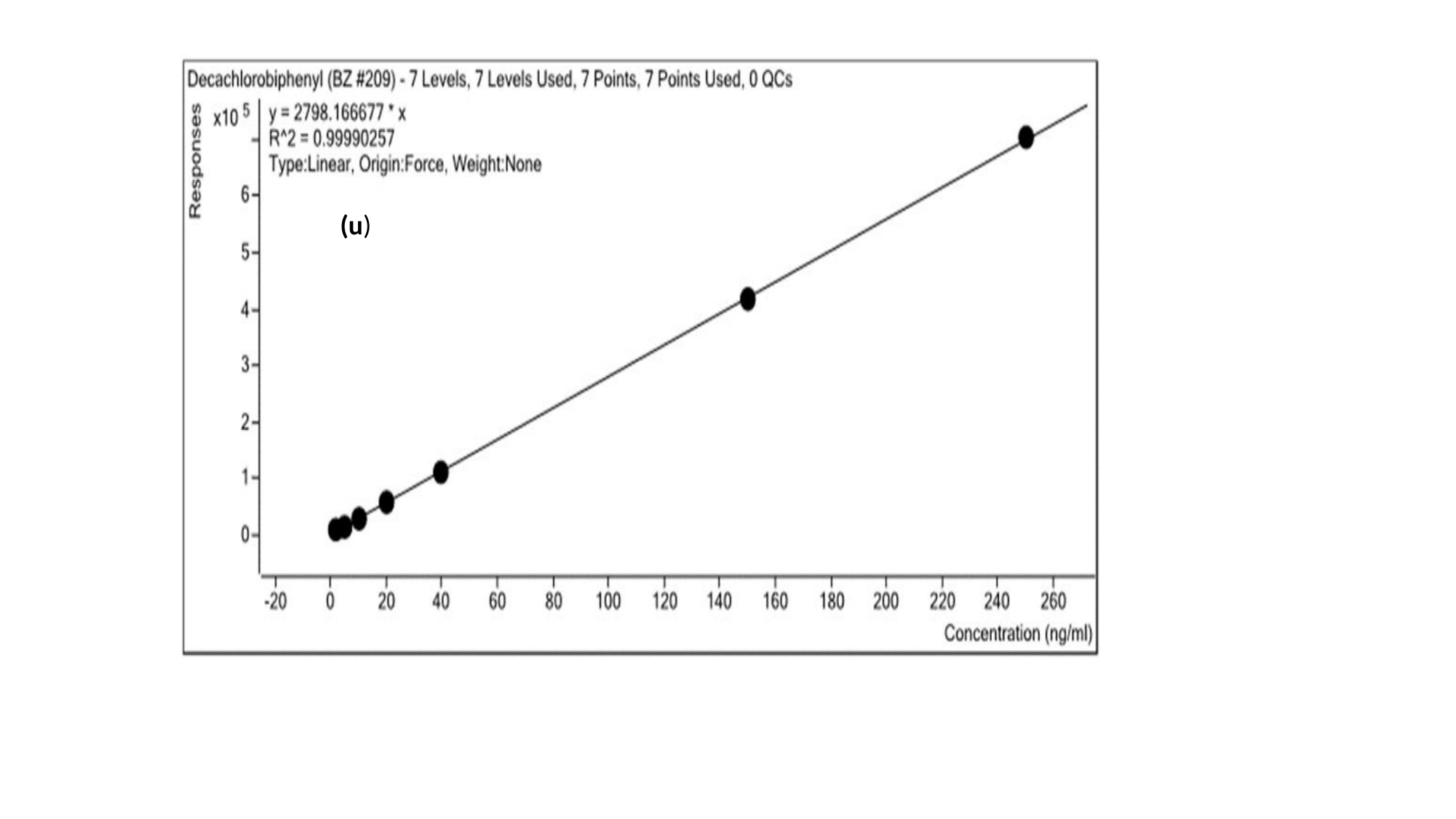

(u)
