# Supplementary material for: Enhanced Detection of Organochlorine Pesticide Residues in Sesame Seeds (Sesamum indicum L.) Using Advanced GC‐MS/MS Techniques
Source: J Anal Methods Chem. 2026 Jan 20;2026:8312847. doi: 10.1155/jamc/8312847 (PMC12817196; doi:10.1155/jamc/8312847)
Supplement: Supplementary file 2 — Supporting Information 2 Supporting Information B. [file JAMC-2026-8312847-s001.pdf]

Figure captions B:

Supplementary informations a-u: GC-MS/MS Chromatograms of 20 CPs in MRM mode

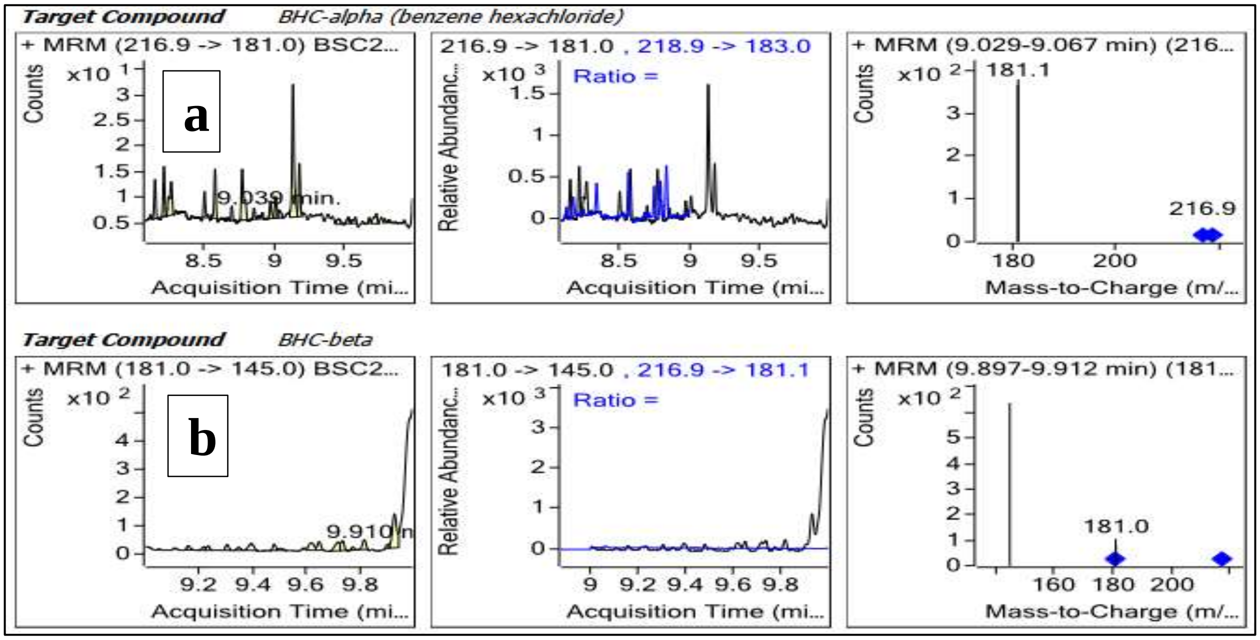

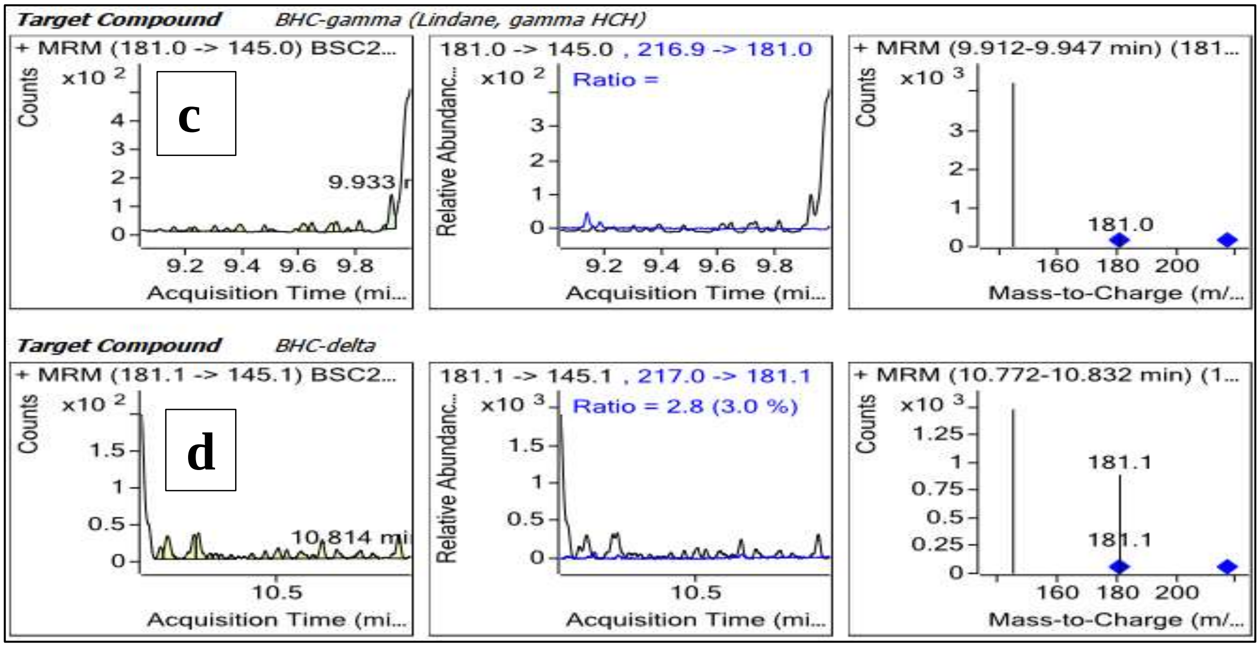

Figures 2a-d. GC-MS/MS Chromatograms of (**a)** α-BHC**, (b)** β- BHC, **(c)** Lindane, (**d)** δ –BHC

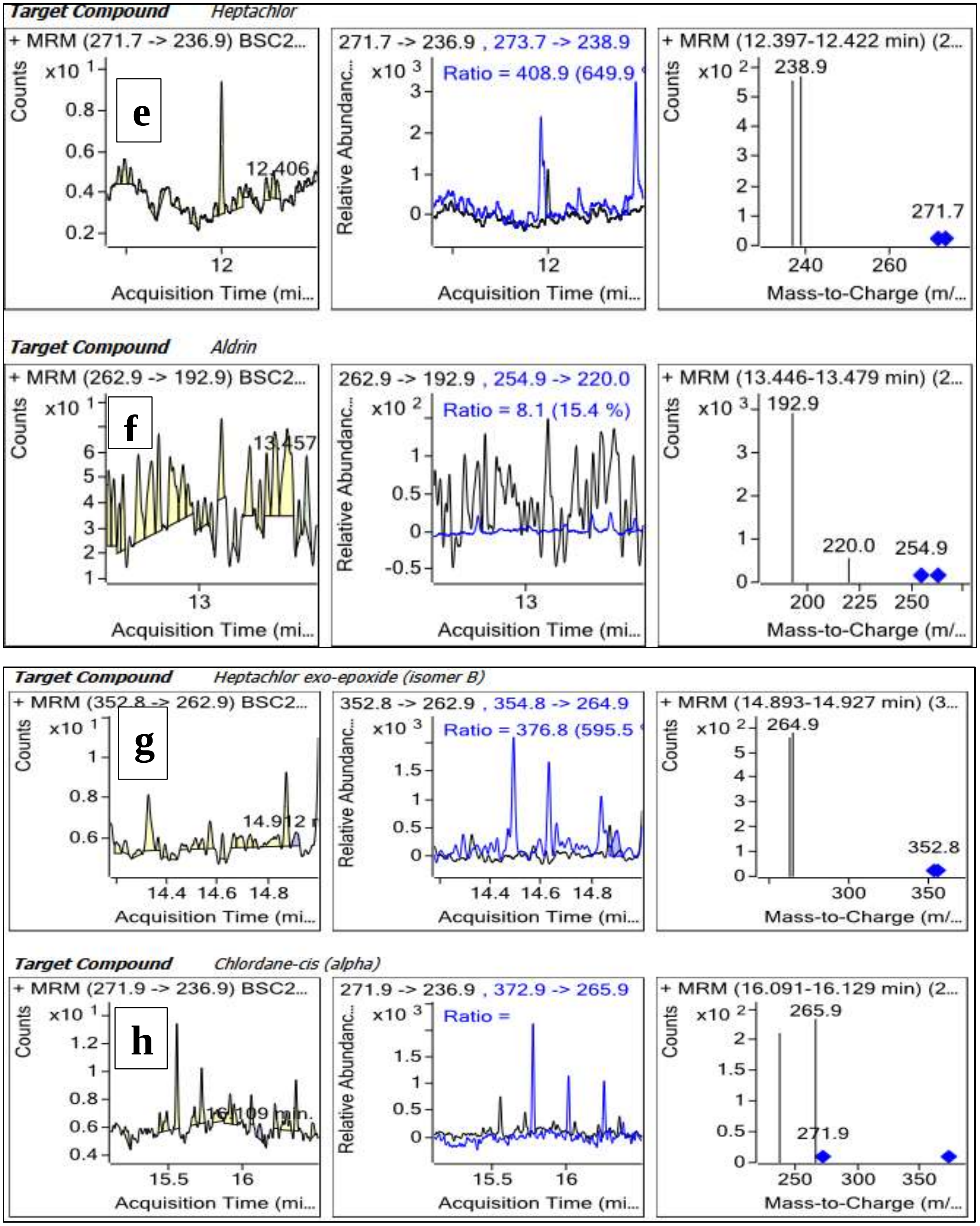

Figures 2e-h. GC-MS/MS Chromatograms of **(e)** Heptachlor (HC),**(f)** Aldrin, **(g)** Heptachlor epoxide(HCE), **(h)** α –Chlordane

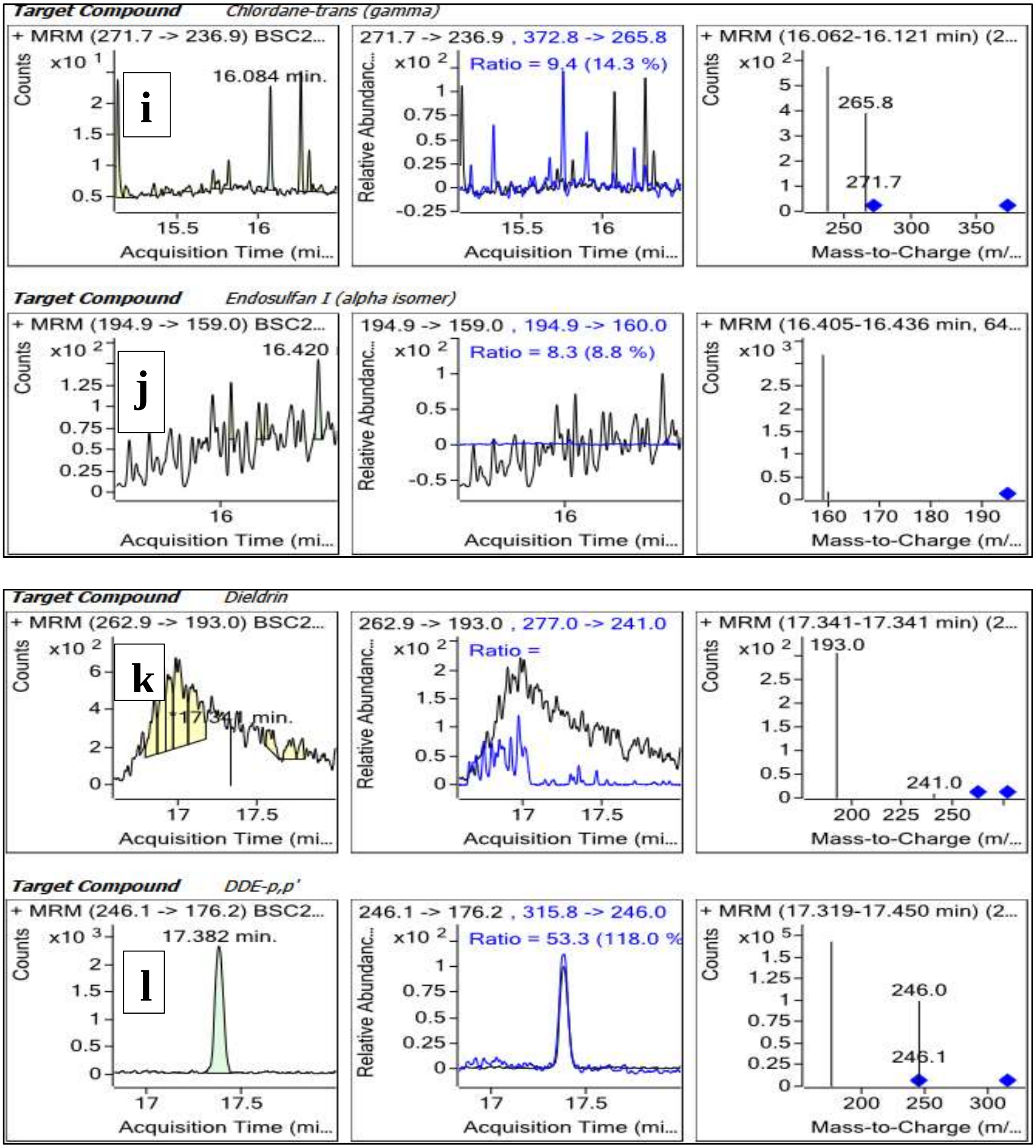

Figures 2i-l. GC-MS/MS Chromatograms of (**i**) γ-Chlordane, (**j**) α-Endosulfan sulfate, **k)** Dieldrin, **l)** DDE-p, p

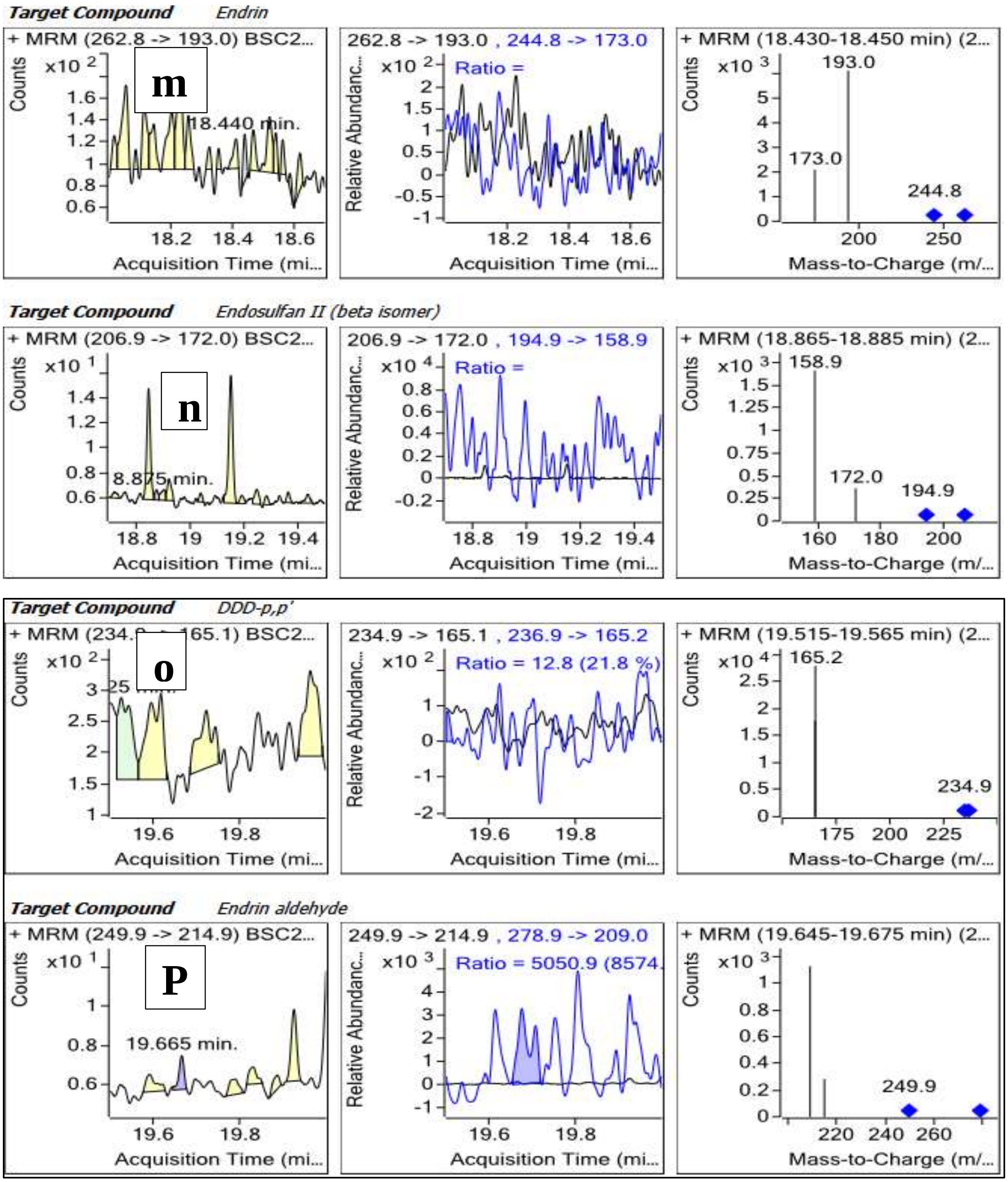

Figures 2m-p. GC-MS/MS Chromatograms of **(m)** Endrin, (**n**) β-Endosulfan sulfate (β-ES), (**o**) DDD, (**p)** Endrin aldehyde

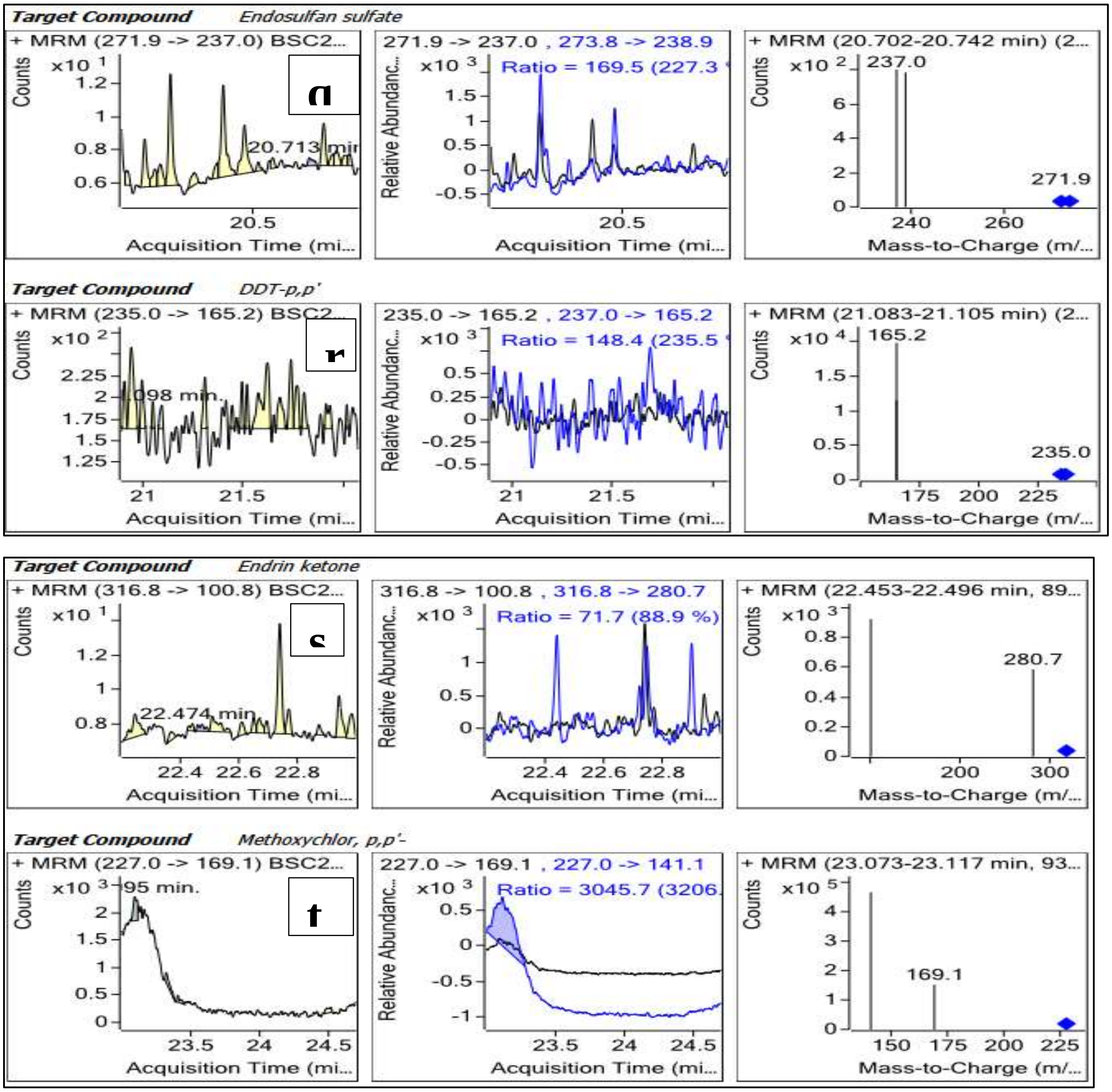

Figures 2q-t.GC-MS/MS Chromatograms of (**q);** Endosulfan sulfate, (**r)** DDT, (**s)** Endrin Ketone, (**t)** Methoxychlor;
